# Performance of waist-to-height ratio as a screening tool for identifying cardiometabolic risk in children: a meta-analysis

**DOI:** 10.1186/s13098-021-00688-7

**Published:** 2021-06-14

**Authors:** Yuan Jiang, Yalan Dou, Hongyan Chen, Yi Zhang, Xiaotian Chen, Yin Wang, Myanca Rodrigues, Weili Yan

**Affiliations:** 1 Department of Clinical Epidemiology and Clinical Trial Unit, National Children’s Medical Center, Children’s Hospital of Fudan University, Shanghai, 201102 People’s Republic of China; 2 Health Research Methodology Graduate Program, Department of Health Research Methods, Evidence, and Impact, McMaster University, Hamilton, ON Canada; 3 Research Unit of Early Intervention of Genetically Related Childhood Cardiovascular Diseases (2018RU002), Chinese Academy of Medical Sciences, Shanghai, China

**Keywords:** Cardiometabolic risk, Diagnostic test, Meta-analysis, Paediatric population, Waist-to-height ratio

## Abstract

**Objective:**

To provide the latest evidence of performance and robustness of waist-to-height ratio (WHtR) in discriminating clusters of cardiometabolic risk factors (CMRs) and promote WHtR in routine primary health care practice in children, a meta-analysis was used.

**Methods:**

Searches was performed in eight databases from inception to July 03, 2020. Inclusion criteria were: (1) observational study, (2) children and adolescents, (3) provided WHtR measurements, (4) had CMRs as outcomes, and (5) diagnostic studies. Exclusion criteria were: (1) non-original articles, (2) unable to extract 2 × 2 contingency tables, (3) not in English or Chinese language, (4) populations comprising clinical patients, or (5) duplicate articles. WHtR cutoff points, 2 × 2 contingency tables were extracted from published reports. Outcomes included: CMR clusters of at least three CMRs (CMR_3_), two (CMR_2_), one (CMR_1_), and CMR components. Bivariate mixed-effects models were performed to estimate the summarised area under the curves (AUSROC) with 95% *CI*s and related indexes. We conducted subgroup analyses by sex and East Asian ethnicity.

**Results:**

Fifty-three observational studies were included. The AUSROC reached 0.91 (95% *CI*: 0.88–0.93), 0.85 (95% *CI*: 0.81, 0.88) and 0.75 (95% *CI*: 0.71, 0.79) for CMR_3_, CMR_2_, and CMR_1_, respectively. The pooled sensitivity and specificity for CMR_3_ reached 0.84 and exceeded 0.75 for CMR_2_. For CMR_1_, the sensitivity achieved 0.55 with 0.84 for specificity. We had similar findings for our subgroup and sensitivity analyses.

**Conclusions:**

WHtR shows good and robust performance in identifying CMRs clustering across racial populations, suggesting its promising utility in public health practice globally.

**Supplementary Information:**

The online version contains supplementary material available at 10.1186/s13098-021-00688-7.

## Background

Childhood obesity is associated with various cardiometabolic risk factors (CMRs), such as elevated fasting blood glucose (FBG), elevated glycated haemoglobin levels, dyslipidaemia, and elevated blood pressure (BP) [1, 2]. The presence of these CMRs may be tracked to adulthood and is associated with an increased risk of cardiovascular and metabolic diseases [3]. Although several behavioural interventions have been developed to curb the obesity epidemic, the long-term success of these interventions remains unsatisfactory [4]. Therefore, identifying the high-risk groups with CMRs from an early age people with obesity could benefit in improving health awareness and behaviour of children and further promote their long-term adherence [5]. This may be of great potential to prevent the development of advanced stages to cardiometabolic diseases.

Overweight or obesity, FBG, lipid profiles, and BP are recommended as important risk factors of cardiovascular diseases in later life [6], indicating current cardiometabolic risk status with clear diagnostic criteria. The abnormal status of which has been included as the component of metabolic syndrome (MetS), as the widely-used definition proposed by the International Diabetes Federation [7]. A blood test is recommended to identify dyslipidaemia among at-risk children [6]. However, it is not feasible to use blood tests to identify CMRs in apparently healthy children based on routine physical examinations, especially in early age children. As the prevention and early detection of CMRs before the onset of obesity-related medical problems are critically important for children, obesity-related anthropometric indexes with easy measurement and classifications have been considered to detect paediatric CMRs rapidly and economically [8, 9].

Waist-to-height ratio (WHtR) was proposed as an index of abdominal obesity that may predict multiple coronary heart disease risk factors in the 1990s [10]. Compared with BMI and WC classifications in defining childhood obesity, WHtR that was the ratio of WC to height shows much less variable with age. This feature makes the classification of obesity or discrimination of CMRs much easier, with a single cutoff across an age range. Previous work has demonstrated that WHtR is an accurate and simple tool for quick and mass screening of CMR in children compared with body mass index (BMI) and waist circumference (WC) [8, 11, 12]. To date, only two meta-analyses have been reported using WHtR for identifying CMRs in the children population, though original studies of this topic are abundant. However, these studies presented limitations with respect to methodology; further, there were only a small number of eligible original studies [13, 14]. The most recent diagnostic meta-analysis in 2016 has demonstrated comparable performances among WHtR, BMI and WC in screening CMRs in children [13]. Of note, the practical application of WC and BMI is inconvenient in the paediatric population because of the requirement for age- and sex-specific references and studies showed that WHtR is age-independent compared with other obesity-related indexes [15–18]. Therefore, WHtR can still be recognised as the most promising one [13, 19] due to good screening accuracy, easy calculation and interpretation.

Moreover, the excellent performance of WHtR with the summarised area under the receiver operating characteristic (ROC) curve (AUC) in discriminating MetS exceeded 0.8 [13]. Since that, a range of new original studies have been published, presenting good but diverse discriminating performances of WHtR in screening various CMRs and MetS based on different population and methodologies [8, 20–24].

A boundary value of WHtR of 0.5 has been proposed for the adult population and widely applied in children studies [25]. The optimal WHtR cutoff for the children population is still inconsistent to achieve satisfactory discrimination of cardiometabolic risk across the studies [21, 26]. Our meta-analysis included different populations with various optimal cutoffs to verify the generalizability and robustness of WHtR for CMRs screening. Through evaluating the performance and robustness of WHtR based on up-to-date studies, our meta-analysis extended previous findings to evaluate the practical values of WHtR as a screening tool in discriminating individual and clusters of CMRs. Furthermore, we reported a range of evaluation indexes for the first time to provide more convincing evidence of the feasibility and applicability of using WHtR in routine public health practices.

## Methods

### Search strategy

Our meta-analysis followed the PRISMA guidelines on a transparent and reproducible process. Comprehensive searches were performed performed using the OVID platform in the following databases: *Pubmed*, *Embase*, *Medline*, *Cochrane Library* and the Chinese databases of *Wan fang*, *CNKI*, *VIP*, and *Sinomed* from inception to July 03, 2020. Search terms included "waist-to-height ratio", "children", and the synonym combinations of them. The search strategy used in PubMed is presented in Additional file 1. The same screening terms were used in the rest of the databases. We also conducted forwards and backwards citation tracking of included studies to identify potentially eligible studies.

### Inclusion criteria

We included studies that met the following criteria: (1) observational in design, (2) had a target population of children and adolescents, or studies of the general population which provided data on a subgroup of children and adolescents, (3) provided data on WHtR measurement, and had (4) at least one of the following CMRs as the primary outcome: elevated FBG, elevated BP, dyslipidaemia, elevated total cholesterol (TC), elevated triglyceride (TG), low high-density leptin cholesterol (HDL-C), elevated low-density leptin cholesterol (LDL-C), central obesity and clustered of CMRs, and (5) diagnostic studies.

### Exclusion criteria

The studies that met the following criteria were excluded: (1) non-original articles, e.g., reviews (2) unable to extract 2 × 2 contingency tables, (3) not in English or Chinese language, (4) populations comprising primarily clinical patients, or (5) duplicate articles.

### Data extraction

To determine study eligibility, two reviewers (YJ & Yl D) independently screened titles, abstracts and identified full-texts. Discrepancies between reviewers were settled through discussions with the reviewer team (Wl Y, Y Z, Xt C, Y W).

These two reviewers extracted the following characteristics of the included studies: author, published year, study year, country, study design, sample size, sex, age, WC measurement technique, WHtR cutoff point, and 2 × 2 contingency table. For those articles without an original contingency table, we extracted the prevalence of CMRs, and the reported diagnostic accuracy parameters (sensitivity, specificity or ROCs) to calculate the value of true-positive (tp), false-positive (fp), true-negative (tn), and false-negative (fn). WHtR was computed as the WC in centimetres divided by the height in centimetres in each original articles.

### Quality assessment

The quality of the included studies was independently assessed by two reviewers (YJ & Yl D) using the quality assessment of diagnostic accuracy studies-2 (QUADAS-2) tool. QUADAS-2 is a revised tool for systematic reviews in evaluating the quality of original diagnostic accuracy studies in the realm of bias and applicability, which involve four domains (patient selection, index test, reference standard, and flow and timing). The reviewer team was in charge of mediating discrepancies regarding quality assessment [27].

### Statistical analysis

We performed the meta-analysis based on five groups of outcomes as follows, referring to the commonly used paediatric MetS definitions of the International Diabetes Federation and the widely studied CMR components in children:

1. Cluster of CMRs

- CMR_3_: presenting with least three of CMRs (elevated FBG, elevated BP, dyslipidaemia, central obesity);
- CMR_2_: presenting with least two of CMRs;
- CMR_1_: presenting with at least one of CMRs.

(2) Elevated FBG
(3) Elevated BP
(4) Dyslipidaemia
  Dyslipidaemia was defined as having at least one of the following abnormalities: elevated TC, or elevated TG, or low HDL-C, or elevated LDL-C. To reduce the heterogeneity of the summarised result, we further pooled dyslipidaemia components separately.
(5) Central obesity

The sensitivity, specificity, positive predictive value (PPV), negative predictive value (NPV), positive likelihood ratio (PLR), negative likelihood ratio (NLR), and prevalence of outcomes were calculated based on tp, fp, tn, and fn extracted from original studies. When results in the original studies were presented by age and sex stratifications, we combined these results into an overall estimation. Summary Area Under Receiver Operating Characteristic (AUSROC), sensitivity, specificity, PLR, NLR, and diagnostic odds ratios (DORs) with 95% confidence intervals (*CI*s) were estimated using the bivariate mixed-effects model based on the random-effects assumption [28]. The advantages of reporting AUSROC lies in its capability of reflecting the interaction between sensitivity and specificity when the threshold effect exists. We assessed between-study heterogeneity using Cochran's *Q*, and also quantified heterogeneity using the *I*^*2*^ statistic. Heterogeneities were substantial when *I*^*2*^ over 50% or *P*-value of *Q* statistic < 0.05. The threshold effects were evaluated by pairwise correlations between sensitivity and 1-specificity with a *P*-value < 0.05.

We further carried out subgroup analyses based on sex and an East Asian ethnicity only due to the limited number of studies in other ethnicities. In our sensitivity analysis, we explored the heterogeneity of the results, for the primary outcome CMR_3_, by only summarising studies that used identical CMR components, e.g. FBG, HDL-C, TG, BP, and central obesity.

Deek's funnel asymmetry tests were used to test publication bias; a non-zero slope coefficient suggested significant bias (*P*-value < 0.05). We sought to figure out possible sources of heterogeneity from diagnostic standards of outcomes, e.g., FBG, BP, dyslipidaemia components, and central obesity, and WHtR cutoff values using meta-regression based on joint models, when we had enough statistical power to do so [29]. The joint model was a comprehensive index in consideration of heterogeneity for sensitivity and specificity. Specifically, the diagnostic criteria for each outcome were classified into a dichotomous variable as follows: elevated FBG ≥ 5.6 mmol/L(100 ng/dL) vs other cutoffs as a group; divided elevated BP into the fixed value of SBP ≥ 130 mmHg or DBP ≥ 85 mmHg or the percentile based on the reference standard; split elevated TG by ≥ 150 mg/dL/ (1.6935 mmol/L) vs other cutoffs as a group; divided low HDL-C into between < 40 mg/dL (0.998 mmol/L) or not; grouped central obesity according to the WC ≥ the 90th percentile of the age- and sex-specific reference standard. The effect of each covariate on sensitivity and specificity separately was not depicted in our meta-regression, as we emphasised the comprehensive index in consideration of heterogeneity from both of them. All analyses were performed using Stata version 15.0 (Stata, Version 15.0 [computer program], Tex Stata Corp., Coll. Station, 2015).

### Role of the funding source

The funding sources had no influence on study design, data collection, analysis, interpretation, paper writing. All authors confirmed that they had full access to all the data in the study and accepted the responsibility to submit for publication.

## Results

**Fig. 1:**
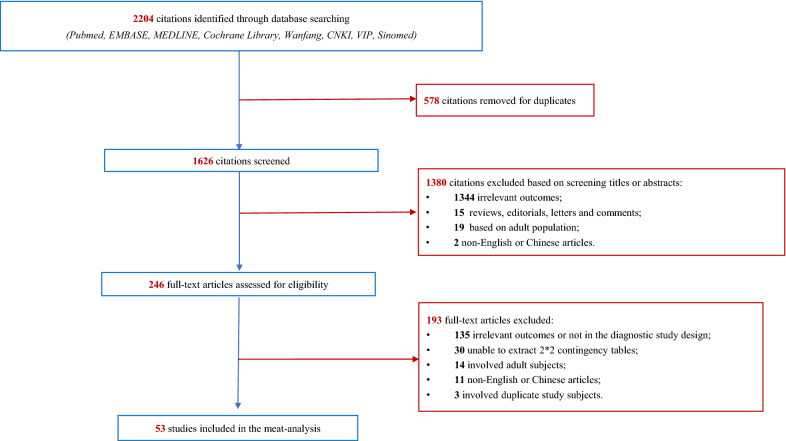
Selection process of primary studies in the meta-analysis. Of the 2204 citations yielded through database searching, 578 citations were removed due to duplicate. In the rest of 1626 citations, 1380 were excluded by screening titles and abstracts. Full-texts were assessed in 246, and an additional of 193 studies further were precluded. Finally, a total of 53 studies were included in the meta-analysis

Figure 1 depicts the search results of this meta-analysis. Of the 2,204 hits, 578 duplicates were removed, and the 1,626 titles and abstracts were screened. Of these, 1,380 were excluded because they were not considered to meet the study inclusion criteria based on the title and abstract. A total of 246 abstracts appeared to be potentially relevant and were collected as full-text articles to be assessed for eligibility for the meta-analysis. A total of 193 articles were subsequently excluded for the reasons listed in Fig. 1, which left 53 studies included in the meta-analysis.

Characteristics of the included studies are presented in Table 1. These studies were performed between 1993 and 2018 and conducted in 24 countries; of these, nine in Europe, seven in Asia, four in South America, three in Africa, and one in North America. Besides, the majority of studies were in the cross-sectional study design, with only one cohort study and one case–control study in our meta-analysis. Study subjects ranged in age from six to 20. The sample size among these studies ranged from 178 to 105,245. WHtR cutoffs extracted contingency tables, and the calculated diagnostic indexes from original studies are listed in Additional file 2: Table S1.

**Table 1:** Characteristics of included studies in the meta-analysis

Author, year	Study year	Country	Design^a^	N(boys/girls)	Age (range or mean ± SD)	CMRs	Categories^b^
Dou [20], 2020	2012–2014	China	1	8130(4325/3805)	7 ~ 18	Elevated FBG; HDL-C; LDL-C; TC; TG; dyslipidaemia; elevated blood pressure; central obesity; CMR_1_; CMR_2_; CMR_3_	1, 2, 3, 4, 5
Nan [42], 2013	NA	China	1	1095	18 ± 0.95	FBG; pre-hypertension; HDL-C; TG; mets (CMR_3_)	1, 2, 3, 4
Hou [43], 2018	2012–2014	China	1	1170	6 ~ 17	FBG; hypertension; HDL-C; TG; 1 RF; 2rfs	1, 2, 3, 4
Perona [44], 2017	NA	Spain	1	1001(468/533)	13.2 ± 1.2	Glucose; SBP hypertension; DBP hypertension; HDL-C; tgs; LDL-C; mets criteria ≥ 3 risks	1, 2, 3, 4
Quadros [45], 2016	2011	Brazil	1	1139	6 ~ 17	Glucose	3
López-González [46], 2016	2011–2015	Mexico	1	365	10 ~ 18	FBG; pre-hypertension; low HDL-C; TG; ≥ 2 rfs	1, 2, 3, 4
Kruger [47], 2013	2003	South African	1	178	14 ~ 18	Glucose; pre-hypertension	2, 3
Xue [48], 2014	2011– 2014	China	1	8378(4245/4133)	6 ~ 17	SBP; DBP; hypertension	3
Motswagole [49], 2011	2000– 2001	South African	1	688(321/367)	9 ~ 15	High BP (95^th^ percentiles)	3
Kromeyer-Hauschild [50], 2013	2003– 2006	Germany	1	6813(3492/3321)	11 ~ 17	Hypertension	3
Chiolero [51], 2013	2005	Switzerland	1	5207	12.3 ± 0.5	Elevated BP	3
Cheah [52], 2018	2015	Malaysia	1	2461(1033/1428)	13 ~ 17	Hypertension	3
Meng [53], 2008	2004	China	2	4939	6 ~ 18	High BP; dyslipidaemia; 1 RF; ≥ 2 rfs; 3rfs	1, 3, 4
Christofaro [54], 2018	2011	Brazil	1	8295	10 ~ 17	Hypertension	3
Ma [55], 2016	1993– 2011	China	1	10,163(5346/4817)	7 ~ 17	Elevated BP	3
Beck [56], 2011	2006	Brazil	1	660(317/343)	14 ~ 19	High BP	3
Wariri [57], 2018	2015	Nigeria	1	367	10 ~ 18	Elevated BP	3
Mishra [58], 2015	2011–2013	India	1	1913	6 ~ 16	High SBP (pre-hypertension); high DBP (pre-hypertension)	3
Liu [59], 2007	2004	China	1	962	5 ~ 19	Dyslipidaemia	4
Zheng [60], 2016	2011–2012	China	1	399(boy only)	9.3 ± 1.7	Dyslipidaemia	4
Chen [61], 2019	2015–2017	China	1	452(255/197)	6 ~ 9	Abdominal fat	5
Ejtahed [62], 2019	2015	Iran	1	14,233(7019/7214)	7 ~ 18	Central obesity	5
Dong [63], 2016	2010	China	1	105,245(60,435/60590)	7 ~ 18	Abdominally overweight	5
Fujita [64], 2011	2008–2010	Japan	1	422(226/196)	10	Abdominal fat	5
Zhou [16], 2014	2010	China	1	16,914(8843/8071)	7 ~ 17	Central obesity; meeting 3 criteria of mets	1, 5
Dai [65], 2014	2009–2010	China	1	18,529(9771/8758)	6 ~ 15	≥ 2 rfs	1
Matsha [66], 2013	2007–2008	South African	1	1272(496/776)	10 ~ 16	2 components of mets	1
Bauer [26], 2015	2006–2009	the United States	1	6052	10 ~ 13	≥ 1 RF; ≥ 2 rfs; ≥ 3 rfs	1
Liu [67], 2015	2006	China	1	3136(1601/1535)	13 ~ 17	Hypertriglyceridemia waist phenotype	1
Seo [21], 2017	2011–2014	Korea	1	2935	10 ~ 19	Mets (CMR_2_)	1
Aguirre [68], 2017	NA	Ecuador	1	395(186/209)	10 ~ 15	Meeting 3 criteria of mets; meeting 4 criteria of mets	1
Adegboye [69], 2010	Denmark 1997–1998, Estonia 1998–1999, Portugal 1999–2000	Denmark, Estonia, Portugal	1	2835(1385/1452)	8.2 ~ 17.3	3 rfs	1
Ma [70], 2017	2006	China	1	3136(1601/1535)	13 ~ 17	Mets (CMR_3_)	1
Zhao [71], 2017	1999–2012	The United States	1	3621(1868/1753)	12 ~ 17	≥ 3 criteria of mets	1
Xu [72], 2017	2007–2011	China	1	11,174(6170/5004)	10 ~ 17	Mets (CMR_3_)	1
Oliveira [73], 2018	2014	Brazil	1	1035(470/565)	12 ~ 20	Mets (CMR_3_)	1
LIU [74], 2017	2010–2011	China	1	928(492/436)	11 ~ 16	Mets (CMR_3_)	1
Arsang-Jang [75], 2019	2003–2016	Iran	1	14,286(7235/7051)	> 10	Mets (CMR_3_)	1
Vasquez [76], 2019	NA	Chile	1	678(354/324)	16	Mets (CMR_3_)	1
Graves [77], 2014	1998–2005	The United Kingdom	3	2856(1368/1488)	7 ~ 13	≥ 3 rfs	1
Tompuri [22], 2019	2007–2009	Finland	1	482(249/233)	6 ~ 8	Meeting 3 criteria of mets	1
Benmohammed [38], 2015	2007	Algeria	1	1088(528/560)	15.5 ± 1.8	Meeting 3 criteria of mets	1
Zhang [78], 2019	NA	China	1	683(366/317)	8–15	Mets (CMR_3_)	1
Yuan [79], 2020	NA	China	1	683(366/317)	8–15	FBG	2
Wang [80], 2019	NA	China	1	683(366/317)	8–15	Hypertension	3
Tee [81], 2020	NA	Malaysia	1	513(211/302)	12–16	Hypertension (90^th^ percentiles, 95^th^ percentiles)	3
Vaquero-Álvarez [82], 2020	2018	Spain	1	265(144/121)	6–16	Hypertension	3
Cristine Silva [24], 2019	NA	Brazil	1	548(238/310)	12–17	Mets (CMR_3_)	1
Li [83], 2020	2013	China	1	15,698(8004/7694)	6–17	Dyslipidaemia, hypertension, CMR_3_	1,3,4
Mai [84], 2020	2014–2015	Vietnam	1	10,936(5537,5399)	6–18	Elevated BP, dyslipidaemia, CMR_3_	1,3,4
Yazdi [85], 2020	2015	Iran	1	14,008(7091,6917)	7–18	Elevated BP, hypertension	3
Kilinc [86], 2019	2011	Turkey	1	2718(1467/1251)	6–17	Abnormality obesity	5
Arellano‐Ruiz [23], 2020	2010	Spain	1	848(408/440)	8–11	HDL-C, TG, elevated BP (95^th^ percentiles), mets	1,3,4

^a^1. Cross-sectional study; 2. Case–control study; 3. Cohort study

^b^1.Clustering of cardiometabolic risk factors; 2. Elevated fasting blood glucose; 3. Elevated blood pressure; 4. Dyslipidaemia; 5. Central obesity;

MetS, metabolic syndrome; CMR: cardiometabolic risk factor; CMR_1_: presenting with least one of CMRs; CMR_2_: presenting with at least two CMRs; CMR_3_: presenting with at least three CMRs; RF, risk factor; FBG: fasting blood glucose; BP, blood pressure; SBP, systolic blood pressure; DBP, diastolic blood pressure; TC, total cholesterol; TG, triglyceride; HDL-C, High-density leptin cholesterol; LDL-C, Low-density leptin cholesterol

**Fig. 2:**
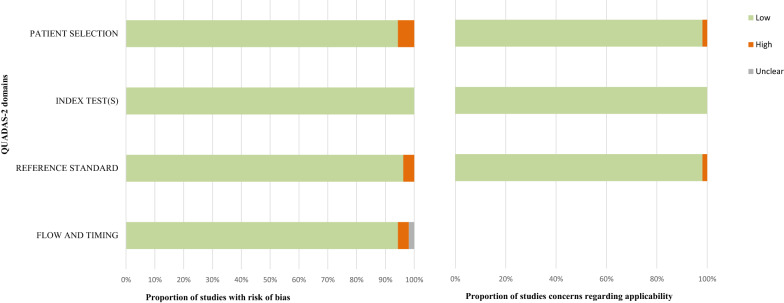
The quality evaluations of included studies through QUADAS-2. The risk of bias was low in most of the studies. Among them, the number of studies having a high risk of bias was two, two, zero and three in the domains of flow and timing, reference standard, index test(s), and patent selection, respectively. The majority of studies were of good applicability. Only one studies presented great concerns in the reference standard domain and one in the patient selection domain

The quality evaluations of the included studies through QUADAS-2 are shown in Additional file 3: Table S2 and Fig. 2. Among 53 included studies, two studies were evaluated in high risk of bias in the domain of flow and timing, two studies with a high probability of bias in the domain of reference standard, zero in the domain of index text(s), and three studies in the domain of patient selection. Moreover, the applicability of original studies was in good performances on the whole, with only one study assessed in the high risk in the domain of reference standard and patient selection, respectively.

Table 2 shows the summarised results of the diagnostic accuracy of WHtR for identifying CMRs. The value of pooled AUSROC increased with the number of CMRs, with 0.91 (95% *CI*: 0.88, 0.93), 0.85(95% *CI*: 0.81, 0.88), and 0.75(95% *CI*: 0.71, 0.79) for CMR_3_, CMR_2_, and CMR_1,_ respectively. For CMR_3_, pooled sensitivity reached to 0.84 (95% *CI*: 0.76, 0.90), specificity of 0.84 (95% *CI*: 0.78, 0.88), and DOR reached 28 (95% *CI*: 14, 54). For single CMR components outcomes, the values of AUSROC were relatively lower in general, with 0.59 (95% *CI*: 0.55, 0.63) for screening elevated FBG, 0.69 (95% *CI*: 0.65, 0.73) for elevated blood pressure, and 0.66 (95% *CI*: 0.62, 0.70) for dyslipidaemia. Notably, the AUSROC reached 0.96 (95% *CI*: 0.94, 0.97) in discriminating central obesity. Threshold effects were founded in pooled elevated FBG, elevated BP, and dyslipidaemia (*P* values < 0.001), with a correlation coefficient of 0.949, 0.801, and 0.859, respectively. Substantial heterogeneities were found among studies in each CMR outcome, which showed that all indexes of *I*^*2*^ were 100%, and *Q* statistics were significant (P < 0.001; Additional file 4: Table S3). No publication bias was founded excepted for elevated blood pressure (P-value = 0.032 based on Deek's funnel plot asymmetry test). The forest plots of pooled sensitivities, specificities, and odd diagnostic ratios in discriminating CMRs are listed (Additional file 5: Figure S1–S14).

**Table 2:** Summarised performance of WHtR screening for CMRs in children and adolescents

Outcome	Population	Prevalence	Threshold effect (correlation coefficient)	*P*-value	AUSROC (95% CI)	Sensitivity (95% CI)	Specificity (95% CI)	DOR (95% CI)
CMR_3_ [16, 20, 22–24, 26, 38, 42, 44, 53, 68–78, 83, 84]	99,331	0.07	− 0.053	0.811	0.91 (0.88, 0.93)	0.84 (0.76, 0.90)	0.84 (0.78, 0.88)	28 (14, 54)
CMR_2_ [20, 21, 26, 43, 46, 53, 65–67]	46,448	0.11	0.271	0.481	0.85 (0.81, 0.88)	0.83 (0.64, 0.93)	0.77 (0.66, 0.84)	16 (6, 45)
CMR_1_ [20, 26, 43, 53]	20,268	0.52	− 0.212	0.788	0.75 (0.71, 0.79)	0.55 (0.43, 0.66)	0.84 (0.74, 0.91)	7 (3, 15)
Elevated FBG [20, 42–47, 79]	13,749	0.21	0.949	< 0.001	0.59 (0.55, 0.63)	0.54 (0.36, 0.70)	0.62 (0.37, 0.82)	2 (1, 3)
Elevated BP [20, 23, 42, 43, 46–57, 80–85]	101,786	0.15	0.801	< 0.001	0.69 (0.65,0.73)	0.55 (0.45,0.64)	0.74 (0.65,0.81)	3 (3,4)
Dyslipidaemia [20, 23, 42–44, 46, 59, 83, 84]	73,092	0.14	0.859	< 0.001	0.66 (0.62,0.70)	0.54 (0.41,0.66)	0.69 (0.60,0.77)	3 (2,3)
Elevated TG [20, 23, 42–44, 46]	12,599	0.08	0.860	0.026	0.70 (0.66, 0.74)	0.58 (0.31, 0.80)	0.71 (0.50, 0.86)	3 (2, 5)
Low HDL-C [20, 23, 42–44, 46]	12,604	0.10	0.930	0.008	0.70 (0.66, 0.74)	0.50 (0.24, 0.77)	0.74 (0.58, 0.86)	3 (2, 5)
Central obesity [16, 20, 61–64, 86]	148,115	0.18	− 0.308	0.502	0.96 (0.94, 0.97)	0.91 (0.84, 0.95)	0.90 (0.86, 0.93)	88 (40, 195)

*WHtR* waist-to-height ratio, *CMR* cardiometabolic risk factor, *CMR*_*3*_ presenting with at least three CMRs, *CMR*_*2*_ presenting with at least two CMRs; *CMR*_*1*_ presenting with least one of CMRs, *FBG* fasting blood glucose, *BP* blood pressure, *TG* triglyceride, *HDL-C* High-density leptin cholesterol, *AUSROC* area under the summary receiver operating characteristic, *DOR* diagnostic odds ratio, *CI* confidence interval

The results of pooled elevated total cholesterol, high low-density leptin cholesterol, and SBP/DBP blood pressure were not reported due to models unstable caused by limited original studies

Subgroup analyses by sex showed similar results with the entire population, with AUSROC of CMR_3_ being 0.89 (95%*CI*: 0.78, 0.95) for boys and 0.84 (0.76, 0.90) for girls, respectively (Additional file 6: Table S4). The sex difference was not significant among each outcome. Performances of WHtR in summarised studies of the East Asian population were consistent with that of the entire population (Additional file 7: Table S5). The AUSROC reached 0.92 (95% *CI*: 0.89, 0.94), 0.88 (95% *CI*: 0.85, 0.90), and 0.79 (95% *CI*: 0.75, 0.82) for CMR_3_, CMR_2_, and CMR_1_, respectively. Notably, the heterogeneity remained high in each outcome of these two subgroups, with *I*^*2*^ ranging from 96 to 100.

We further performed the sensitivity analysis for CMR_3_ in 13 studies with five identical components: elevated FBG, low HDL-C, elevated TG, elevated BP, and central obesity. We found that the AUSROC reached 0.90 (95%*CI*: 0.80, 0.95) with the sensitivity of 0.89 (95%*CI*: 0.82, 0.93) and specificity of 0.89 (95%*CI*: 0.82, 0.93). Although the threshold effect was not significant (p = 0.905), the heterogeneity remained large, with *I*^*2*^ of 100% (Additional file 8: Table S6). We did not analyse the other outcomes, e.g., CMR_2_ or CMR_1_, because the numbers of studies with identical components were less than three.

In our meta-regression analyses (Table 3), we found that various WHtR cutoffs used in the original studies may account for 69% to 99% of the heterogeneity of the meta-analyses, while the differences in using different diagnostic criteria for defining components of CMRs did not make a significant contribution.

**Table 3:** Possible sources of heterogeneity: meta-regressions

	Standard of Diagnosis			WHtR cut-off		
Outcome	χ^2^	*P*	*I*^*2*^	χ^2^	*P*	*I*^*2*^
CMR_3_	NA	NA	NA	298.64	< 0.001	99 (99–100)
CMR_2_	NA	NA	NA	54.92	< 0.001	96 (94–99)
CMR_1_	NA	NA	NA	6.51	0.04	69 (31–100)
Elevated FBG	2.59	0.27	23 (0–100)	19.03	< 0.001	89 (79–100)
Elevated BP	1.46	0.48	0 (0–100)	169.46	< 0.001	99 (98–99)
Dyslipidaemia	NA	NA	NA	40.33	< 0.001	95 (91–99)
Elevated TG	2.84	0.24	30 (0–100)	6.7	0.04	70 (33–100)
Low HDL-C	3.05	0.22	35 (0–100)	9.89	0.01	80 (56–100)
Central obesity	1.77	0.47	0 (0–100)	1.34	0.51	0 (0–100)

*NA* not applicable, *WHtR* waist-to-height ratio, *CMR* cardiometabolic risk factor, *CMR*_*3*_ presenting with at least three CMRs, *CMR*_*2*_ presenting with at least two CMRs, *CMR*_*1*_ presenting with least one of CMRs, *FBG* fasting blood glucose; *BP* blood pressure, *TG* triglyceride, *HDL-C* High-density leptin cholesterol; The results of elevated total cholesterol, high low-density leptin cholesterol, and SBP/DBP blood pressure were not reported

## Discussion

In this study, the excellent performances of WHtR significantly prove its advantage in screening children with CMRs in routine practice. We found that the satisfactory performance of WHtR was notable for discriminating clustered CMRs. Especially for identifying CMR_3_ (affected with at least three CMR abnormalities), the AUSROC would reach 0.91 with both the sensitivity and specificity achieved 0.84, and the DOR of 28 indicated that the odds of being affecting CMR_3_ were 28 times higher among children with WHtR over cutoff values than that of non-CMR_3_. Besides, the AUSROC attained 0.85 for screening CMR_2_ and 0.75 for CMR_1_. As a comprehensive concept with public health significance, the clustered CMRs can be detected by WHtR accurately, and the more severe the CMR, the easier it will be identified. Moreover, the summarised results posed that WHtR remained robust in East Asian populations and each sex. These findings further strengthen the evidence of the application values of WHtR and promote this quick and convenient measurement as a routine screening tool in the practice of prevention and control of CMR in children.

After the meta-analysis previously published in 2016 [13], many recent original studies involving more diverse ethnicities and methodologies were summarised in our meta-analysis through the more optimised statistical models, which further provided superior and more informative findings. Our meta-analysis of 23 studies presented a slightly better performance (AUSROC of 0.91) for CMR_3_ screening compared with the previous one (AUSROC = 0.81, 95% *CI*: 0.77, 0.86) that identified MetS [13]. Furthermore, the consistent performance of WHtR in screening cardiometabolic diseases has been demonstrated in adult populations based on a meta-analysis [17], with the pooled AUC of WHtR for incident diabetes, MetS, and total incident cardiovascular disease were all over 0.7. These findings suggest that WHtR could be a useful tool in identifying both CMR in children and cardiometabolic diseases in adults.

Central obesity is a critical component and precondition of MetS in children and adolescents [30], which can be defined by the sex and age-specific WC percentile for children [31]. As WHtR had a substantial accuracy in identifying central obesity with the excellent AUSROC of 0.96, the criterion for clustered CMRs including the central obesity in our study may make the accuracy of identification of clustered results slightly higher (the AUSROC reached 0.91for CMR_3_), which has been indicated in our previous study (AUC for CMR_3_ including central obesity v.s. that excluding central obesity, 0.89 v.s 0.76) [18]. Early evidence also supported the good performance of WHtR for screening CMR components excluded central obesity. Previous meta-analyses of the paediatric population illustrated that the pooled AUC of WHtR screening MetS factors without central obesity reached 0.71 (95% *CI*: 0.66, 0.75) [12], and for the cardiometabolic comorbidities of at least three items was 0.69 (95% *CI*: 0.57, 0.80), which were acceptably accurate [13]. CMRs such as obesity, hypertension, and dyslipidaemia tend to cluster in youth [32]. Cohort studies show strong evidence that single or cluster CMRs present during adolescence likely track forward to adulthood and are related to markedly increased risk for cardiovascular or metabolic disease [33–37]. These all underline the importance of identifying children at increased risk for cardiometabolic comorbidity as early in life as possible.

We found that our meta-analyses suffer from high heterogeneity. Although WHtR over 0.5 has been proposed as a healthy cutoff for avoiding cardiovascular disease and diabetes for adults and been widely used [11], various WHtR cutoffs have been adopted in the original studies to fit their study populations with different prevalence levels of CMR. An Algerian study showed that the optimal cutoff value of 0.55 achieved a specificity of 0.89 in identifying girls with MetS, while this number was only 0.75 using the well-reported cutoff of 0.5 [38]. Our previous study also suggested that the critical value of WHtR of 0.467 is more accurate than 0.50 in Chinese children [20]. We used the AUSROC to minimise the influence of the threshold effects [17, 30]. The findings from our meta-regression demonstrated that heterogeneity of our findings might likely be explained by different threshold cutoffs used to classify WHtR and less likely due to different standards of diagnosing outcome variables, e.g., slightly different diagnosing cutoffs. In addition, subgroup analyses for sex and ethnic background, i.e. Eastern Asian populations, as well as our sensitivity analyses summarising studies with homogenous CMR components also supported the robustness of the main results. Moreover, the biases were limited in included original studies among all four domains by QUADAS-2. Although most of the studies did not report whether blinding was used, we still believe that biases from unblinding do not exist in our study, because both WHtR and the standard of CMRs were defined based on objectively measured numerical values. It is not likely to bias the quality evaluation results.

Our meta-analysis had several strengths. First, our meta-analysis based on the most updated, relevant studies over the world provided the most substantial evidence to date about WHtR as a promising screening tool for CMRs in the children population. Second, our study presented multiple summary diagnostic accuracy indexes, including AUSROC, sensitivity, specificity, DOR, and likelihood ratio, which were more informative for public-health practice decisions [28]. Third, we presented WHtR performance for a series of a single component of CMRs, providing decision-makers with thorough information when using it for populations with different disease status. We further discriminated the summary results in three levels of CMR clusters representing the comprehensive cardiometabolic risk in children. Fourth, this was the first meta-analysis discriminating the performance of WHtR for CMRs in the East Asia population who were less tolerable of obesity and developed earlier to CMRs at a lower level of BMI [39].

Our analyses must be interpreted in the context of the limitations of the available data. First, we found significant heterogeneity of included studies and using diverse WHtR cutoffs for screening CMRs as the main determinant. Overall, the results of analyses for meta-regressions, subgroup analyses, and sensitivity analyses into considerations highlighted the robustness and the accuracy of WHtR in screening CMRs. Additionally, our synthesised results were more evidence-based than a single original study. The variation in measuring and defining WHtR and CMRs existed in the original studies, and our evidence-based synthesis to some extent helped ensure the generalizability of the main findings for translation into clinical practice. Secondly, we found fewer relevant studies from other races, such as Caucasians and Africans; therefore, the performance of WHtR in these populations needed more evidence so that we may be able to accurately generalise to these populations. Third, obesity-related CMRs vary with age; given the lack of this information from the original studies included in our review, we could not consider this factor in our analyses. Fourth, the different WC measurement techniques may cause bias and variability among original studies [40] and lead to the heterogeneity of our meta-analysis. However, a systematic review of 120 studies suggested that different WC measurement techniques are not likely to bias the association between the WC or WHtR and cardiometabolic diseases [41]. Future studies and efforts are expected to translate this evidence into practise of preventing and controlling CMRs in children and adolescents.

## Conclusion

In summary, our meta-analysis presents a good performance of WHtR in identifying children and adolescents with CMRs, and the findings appear robust to other factors, including ethnicity. This evidence strongly supports WHtR as a promising and practical tool in routine primary health care practice of controlling obesity-related CMRs in children and adolescents.

## Supplementary Information

**Additional file 1.** The search strategy used in PubMed of this work.

**Additional file 2: Table S1.** Results of diagnostic test extracting from eligible original articles.

**Additional file 3: Table S2.** Evaluation of the risk of bias and applicability of included studies by QUADAS-2.

**Additional file 4: Table S3.** Other pooled results of WHtR screening for CMRs in children and adolescents.

**Additional file 5: Figure S1-S14.** The forest plots of pooled sensitivities, specificities, and odd diagnostic ratios in discriminating CMRs.

**Additional file 6: Table S4.** Pooled results of WHtR screening for CMRs by sex in children and adolescents.

**Additional file 7: Table S5.** Pooled results of WHtR screening for CMRs in East-Asian children.

**Additional file 8: Table S6.** Sensitivity analyses: pooled results of WHtR screening for clusters of CMR with identical components

## Data Availability

The datasets used and/or analysed during the current study are available from the corresponding author on reasonable request.

## Abbreviations

AUC: Area under the curve
AUSROC: Summary Area Under Receiver Operating Characteristic
BMI: Body mass index
BP: Blood pressure
CIs: Confidence intervals
CMRs: Cardiometabolic risk factors
DOR: Diagnostic odds ratio
FBG: Fasting blood glucose
HDL-C: High-density leptin cholesterol
LDL-C: Low-density leptin cholesterol
MetS: Metabolic syndrome
NLR: Negative likelihood ratio
NPV: Negative predictive value
PLR: Positive likelihood ratio
PPV: Positive predictive value
QUADAS-2: Quality assessment of diagnostic accuracy studies-2
ROC: Receiver operating characteristic curve
TC: Total cholesterol
TG: Triglyceride
WC: Waist circumference
WHtR: Waist-to-height ratio
