# Supplementary material for: Performance of waist-to-height ratio as a screening tool for identifying cardiometabolic risk in children: a meta-analysis
Source: Diabetol Metab Syndr. 2021 Jun 14;13:66. doi: 10.1186/s13098-021-00688-7 (PMC8201900; doi:10.1186/s13098-021-00688-7)
Supplement: Supplementary file 1 — Additional file 1. The search strategy used in PubMed of this work. [file 13098_2021_688_MOESM1_ESM.docx]

**The search strategy used in PubMed**

("W-HtR" or "WC/height" or "WHtR" or "waist: height ratio" or "waist-to-height ratio" or "waist to height ratio" or "wthr" or "Waist-Height Ratio" [Mesh] or "Waist-to-height" or "waist to height" or "waist height" or "waist circumference to height" or "Height Weight Ratio") AND ("child" [MeSH Terms] or "adolescent" [MeSH Terms] or adolescen* or teen* or youth* or children).

The same screening terms were used in the rest of the databases. We also conducted forwards and backwards citation tracking of included studies.
