# Supplementary material for: Performance of waist-to-height ratio as a screening tool for identifying cardiometabolic risk in children: a meta-analysis
Source: Diabetol Metab Syndr. 2021 Jun 14;13:66. doi: 10.1186/s13098-021-00688-7 (PMC8201900; doi:10.1186/s13098-021-00688-7)
Supplement: Supplementary file 2 — Additional file 2: Table S1. Results of diagnostic test extracting from eligible original articles. [file 13098_2021_688_MOESM2_ESM.docx]

**Table S1. Results of diagnostic test extracting from eligible original articles**

| Author | N | Sex | CMR (Outcomes) | Outcomes Categories^a^ | Waist circumference measurement | WHtR  cut-off | tp | fp | fn | tn | Sensitivity | Specificity | PPV | NPV | Prevalence | PLR | NLR |
| --- | --- | --- | --- | --- | --- | --- | --- | --- | --- | --- | --- | --- | --- | --- | --- | --- | --- |
| Dou YL(1), 2020 | 8130 | both | CMR_1_ | 1 | 1cm above the umbilicus | 0.467 | 2073 | 232 | 2457 | 3368 | 0.46 | 0.94 | 0.90 | 0.58 | 0.557 | 7.10 | 0.58 |
|  | 4325 | boy | CMR_1_ | 1 |  | 0.481 | 1209 | 96 | 1379 | 1641 | 0.47 | 0.94 | 0.93 | 0.54 | 0.598 | 8.45 | 0.56 |
|  | 3805 | girl | CMR_1_ | 1 |  | 0.456 | 836 | 134 | 1106 | 1729 | 0.43 | 0.93 | 0.86 | 0.61 | 0.510 | 5.99 | 0.61 |
|  | 8130 | both | CMR_2_ | 1 |  | 0.467 | 1317 | 988 | 506 | 5319 | 0.72 | 0.84 | 0.57 | 0.91 | 0.224 | 4.61 | 0.33 |
|  | 4325 | boy | CMR_2_ | 1 |  | 0.481 | 849 | 456 | 312 | 2708 | 0.73 | 0.86 | 0.65 | 0.90 | 0.268 | 5.07 | 0.31 |
|  | 3805 | girl | CMR_2_ | 1 |  | 0.456 | 457 | 513 | 205 | 2630 | 0.69 | 0.84 | 0.47 | 0.93 | 0.174 | 4.23 | 0.37 |
|  | 8130 | both | CMR_3_ | 1 |  | 0.467 | 434 | 1871 | 59 | 5766 | 0.88 | 0.76 | 0.19 | 0.99 | 0.061 | 3.59 | 0.16 |
|  | 4325 | boy | CMR_3_ | 1 |  | 0.481 | 290 | 1015 | 41 | 2979 | 0.88 | 0.75 | 0.22 | 0.99 | 0.077 | 3.45 | 0.17 |
|  | 3805 | girl | CMR_3_ | 1 |  | 0.456 | 138 | 832 | 24 | 2811 | 0.85 | 0.77 | 0.14 | 0.99 | 0.043 | 3.73 | 0.19 |
|  | 8123 | both | elevated FBG | 2 |  | 0.467 | 732 | 1566 | 1530 | 4295 | 0.32 | 0.73 | 0.32 | 0.74 | 0.278 | 1.21 | 0.92 |
|  | 4321 | boy | elevated FBG | 2 |  | 0.481 | 445 | 856 | 889 | 2131 | 0.33 | 0.71 | 0.34 | 0.71 | 0.309 | 1.16 | 0.93 |
|  | 3802 | girl | elevated FBG | 2 |  | 0.456 | 266 | 701 | 662 | 2173 | 0.29 | 0.76 | 0.28 | 0.77 | 0.244 | 1.18 | 0.94 |
|  | 8129 | both | elevated BP | 3 |  | 0.467 | 703 | 1601 | 572 | 5253 | 0.55 | 0.77 | 0.31 | 0.90 | 0.157 | 2.36 | 0.59 |
|  | 4324 | boy | elevated BP | 3 |  | 0.481 | 472 | 832 | 357 | 2663 | 0.57 | 0.76 | 0.36 | 0.88 | 0.192 | 2.39 | 0.57 |
|  | 3805 | girl | elevated BP | 3 |  | 0.456 | 218 | 752 | 228 | 2607 | 0.49 | 0.78 | 0.22 | 0.92 | 0.117 | 2.18 | 0.66 |
|  | 8126 | both | HDL-C | 4 |  | 0.467 | 288 | 2017 | 343 | 5478 | 0.46 | 0.73 | 0.12 | 0.94 | 0.078 | 1.70 | 0.74 |
|  | 4322 | boy | HDL-C | 4 |  | 0.481 | 188 | 1117 | 191 | 2826 | 0.50 | 0.72 | 0.14 | 0.94 | 0.088 | 1.75 | 0.70 |
|  | 3804 | girl | HDL-C | 4 |  | 0.456 | 89 | 881 | 163 | 2671 | 0.35 | 0.75 | 0.09 | 0.94 | 0.066 | 1.42 | 0.86 |
|  | 8120 | both | LDL-C | 4 |  | 0.467 | 138 | 2165 | 145 | 5672 | 0.49 | 0.72 | 0.06 | 0.98 | 0.035 | 1.77 | 0.71 |
|  | 4320 | boy | LDL-C | 4 |  | 0.481 | 80 | 1223 | 58 | 2959 | 0.58 | 0.71 | 0.06 | 0.98 | 0.032 | 1.98 | 0.59 |
|  | 3800 | girl | LDL-C | 4 |  | 0.456 | 57 | 913 | 88 | 2742 | 0.39 | 0.75 | 0.06 | 0.97 | 0.038 | 1.57 | 0.81 |
|  | 8123 | both | TC | 4 |  | 0.467 | 142 | 2160 | 245 | 5576 | 0.37 | 0.72 | 0.06 | 0.96 | 0.048 | 1.31 | 0.88 |
|  | 4320 | boy | TC | 4 |  | 0.481 | 89 | 1213 | 106 | 2912 | 0.46 | 0.71 | 0.07 | 0.96 | 0.045 | 1.55 | 0.77 |
|  | 3803 | girl | TC | 4 |  | 0.456 | 53 | 917 | 139 | 2694 | 0.28 | 0.75 | 0.05 | 0.95 | 0.050 | 1.09 | 0.97 |
|  | 8124 | both | TG | 4 |  | 0.467 | 255 | 2045 | 188 | 5636 | 0.58 | 0.73 | 0.11 | 0.97 | 0.055 | 2.16 | 0.58 |
|  | 4320 | boy | TG | 4 |  | 0.481 | 162 | 1139 | 88 | 2931 | 0.65 | 0.72 | 0.12 | 0.97 | 0.058 | 2.32 | 0.49 |
|  | 3804 | girl | TG | 4 |  | 0.456 | 93 | 876 | 100 | 2735 | 0.48 | 0.76 | 0.10 | 0.96 | 0.051 | 1.99 | 0.68 |
|  | 8130 | both | dyslipidaemia | 4 |  | 0.467 | 615 | 1690 | 754 | 5071 | 0.45 | 0.75 | 0.27 | 0.87 | 0.168 | 1.80 | 0.73 |
|  | 4325 | boy | dyslipidaemia | 4 |  | 0.481 | 378 | 927 | 371 | 2649 | 0.50 | 0.74 | 0.29 | 0.88 | 0.173 | 1.95 | 0.67 |
|  | 3805 | girl | dyslipidaemia | 4 |  | 0.456 | 228 | 742 | 392 | 2443 | 0.37 | 0.77 | 0.24 | 0.86 | 0.163 | 1.58 | 0.82 |
|  | 8130 | both | central obesity | 5 |  | 0.467 | 1854 | 451 | 172 | 5653 | 0.92 | 0.93 | 0.80 | 0.97 | 0.249 | 12.39 | 0.09 |
|  | 4325 | boy | central obesity | 5 |  | 0.481 | 1117 | 188 | 121 | 2899 | 0.90 | 0.94 | 0.86 | 0.96 | 0.286 | 14.82 | 0.10 |
|  | 3805 | girl | central obesity | 5 |  | 0.456 | 733 | 237 | 55 | 2780 | 0.93 | 0.92 | 0.76 | 0.98 | 0.207 | 11.84 | 0.08 |
| NAN ZH(2), 2013 | 1095 | both | MetS (CMR_3_) | 1 | The midpoint of the upper edge of the ilium muscle and the lower edge of the ribs | 0.46 | 44 | 22 | 72 | 957 | 0.38 | 0.98 | 0.67 | 0.93 | 0.106 | 16.88 | 0.63 |
|  | 1095 | both | FBG | 2 |  | 0.46 | 30 | 36 | 73 | 956 | 0.29 | 0.96 | 0.45 | 0.93 | 0.094 | 8.03 | 0.74 |
|  | 1095 | both | prehypertension | 3 |  | 0.46 | 34 | 32 | 194 | 835 | 0.15 | 0.96 | 0.52 | 0.81 | 0.208 | 4.04 | 0.88 |
|  | 1095 | both | HDL-C | 4 |  | 0.46 | 6 | 60 | 77 | 952 | 0.07 | 0.94 | 0.09 | 0.93 | 0.076 | 1.22 | 0.99 |
|  | 1095 | both | TGs | 4 |  | 0.46 | 10 | 56 | 80 | 949 | 0.11 | 0.94 | 0.15 | 0.92 | 0.082 | 1.99 | 0.94 |
| HOU YP(3), 2018 | 1170 | both | 1 RF | 1 | 1cm above the umbilicus | 0.5 | 223 | 178 | 273 | 496 | 0.45 | 0.74 | 0.56 | 0.64 | 0.424 | 1.70 | 0.75 |
|  | 1154 | both | 2RFs | 1 |  | 0.5 | 112 | 258 | 65 | 719 | 0.63 | 0.74 | 0.30 | 0.92 | 0.104 | 2.39 | 0.50 |
|  | 1170 | both | FPG | 2 |  | 0.5 | 161 | 794 | 26 | 189 | 0.86 | 0.19 | 0.17 | 0.88 | 0.160 | 1.07 | 0.72 |
|  | 1170 | both | hypertension | 3 |  | 0.5 | 133 | 306 | 87 | 644 | 0.60 | 0.68 | 0.30 | 0.88 | 0.188 | 1.88 | 0.58 |
|  | 1170 | both | HDL-C | 4 |  | 0.5 | 82 | 268 | 81 | 739 | 0.50 | 0.73 | 0.23 | 0.90 | 0.139 | 1.89 | 0.68 |
|  | 1170 | both | TG | 4 |  | 0.5 | 51 | 326 | 27 | 766 | 0.65 | 0.70 | 0.14 | 0.97 | 0.067 | 2.19 | 0.49 |
| Perona JS(4), 2017 | 468 | boy | glucose | 2 | The midpoint of the upper edge of the ilium muscle and the lower edge of the ribs | 0.44 | 18 | 208 | 16 | 226 | 0.53 | 0.52 | 0.08 | 0.93 | 0.073 | 1.10 | 0.90 |
|  | 533 | girl | glucose | 2 |  | 0.44 | 52 | 209 | 46 | 226 | 0.53 | 0.52 | 0.20 | 0.83 | 0.184 | 1.10 | 0.90 |
|  | 468 | boy | SBP hypertension | 3 |  | 0.46 | 26 | 108 | 8 | 326 | 0.76 | 0.75 | 0.19 | 0.98 | 0.073 | 3.07 | 0.31 |
|  | 533 | girl | SBP hypertension | 3 |  | 0.46 | 75 | 109 | 24 | 325 | 0.76 | 0.75 | 0.41 | 0.93 | 0.186 | 3.02 | 0.32 |
|  | 468 | boy | DBP hypertension | 3 |  | 0.47 | 23 | 139 | 11 | 295 | 0.68 | 0.68 | 0.14 | 0.96 | 0.073 | 2.11 | 0.48 |
|  | 533 | girl | DBP hypertension | 3 |  | 0.47 | 66 | 139 | 32 | 296 | 0.67 | 0.68 | 0.32 | 0.90 | 0.184 | 2.11 | 0.48 |
|  | 468 | boy | HDL-C | 4 |  | 0.45 | 19 | 182 | 15 | 252 | 0.56 | 0.58 | 0.09 | 0.94 | 0.073 | 1.33 | 0.76 |
|  | 533 | girl | HDL-C | 4 |  | 0.45 | 56 | 183 | 42 | 252 | 0.57 | 0.58 | 0.23 | 0.86 | 0.184 | 1.36 | 0.74 |
|  | 468 | boy | TG | 4 |  | 0.44 | 19 | 217 | 15 | 217 | 0.56 | 0.50 | 0.08 | 0.94 | 0.073 | 1.12 | 0.88 |
|  | 533 | girl | TG | 4 |  | 0.44 | 55 | 217 | 43 | 218 | 0.56 | 0.50 | 0.20 | 0.84 | 0.184 | 1.13 | 0.88 |
|  | 468 | boy | LDL-C | 4 |  | 0.45 | 19 | 195 | 15 | 239 | 0.56 | 0.55 | 0.09 | 0.94 | 0.073 | 1.24 | 0.80 |
|  | 533 | girl | LDL-C | 4 |  | 0.45 | 54 | 196 | 44 | 239 | 0.55 | 0.55 | 0.22 | 0.84 | 0.184 | 1.22 | 0.82 |
|  | 468 | boy | MetS criteria≥3 risks | 1 |  | 0.56 | 31 | 17 | 3 | 417 | 0.91 | 0.96 | 0.65 | 0.99 | 0.073 | 23.28 | 0.09 |
|  | 533 | girl | MetS criteria≥3 risks | 1 |  | 0.56 | 89 | 17 | 9 | 418 | 0.91 | 0.96 | 0.84 | 0.98 | 0.184 | 23.24 | 0.10 |
| Quadros TM(5), 2016 | 1139 | both | glucose | 2 | 1cm above the umbilicus | 0.5 | 24 | 203 | 52 | 860 | 0.32 | 0.81 | 0.11 | 0.94 | 0.067 | 1.65 | 0.85 |
| López-González D(6), 2016 | 361 | both | ≥2 RFs | 1 |  | 0.5 | 116 | 156 | 4 | 85 | 0.97 | 0.35 | 0.43 | 0.96 | 0.332 | 1.49 | 0.09 |
|  | 360 | both | FBG | 2 |  | 0.5 | 29 | 248 | 10 | 73 | 0.74 | 0.23 | 0.10 | 0.88 | 0.108 | 0.96 | 1.13 |
|  | 358 | both | prehypertension | 3 |  | 0.5 | 28 | 245 | 0 | 85 | 1.00 | 0.26 | 0.10 | 1.00 | 0.078 | 1.35 | 0.00 |
|  | 364 | both | LHDL-C | 4 |  | 0.5 | 191 | 84 | 15 | 74 | 0.93 | 0.47 | 0.69 | 0.83 | 0.566 | 1.74 | 0.16 |
|  | 362 | both | TG | 4 |  | 0.5 | 129 | 149 | 8 | 76 | 0.94 | 0.34 | 0.46 | 0.90 | 0.378 | 1.42 | 0.17 |
| Kruger HS(7), 2013 | 178 | both | glucose | 2 | The midpoint of the upper edge of the ilium muscle and the lower edge of the ribs | 0.4 | 14 | 99 | 3 | 62 | 0.82 | 0.39 | 0.12 | 0.95 | 0.096 | 1.34 | 0.46 |
|  | 178 | both | prehypertension | 3 |  | 0.41 | 13 | 79 | 7 | 79 | 0.65 | 0.50 | 0.14 | 0.92 | 0.112 | 1.30 | 0.70 |
| Xue J(8), 2014 | 4245 | boy | SBP hypertension | 3 | 1cm above the umbilicus | 0.49 | 517 | 699 | 336 | 2693 | 0.61 | 0.79 | 0.43 | 0.89 | 0.201 | 2.94 | 0.50 |
|  | 4133 | girl | SBP hypertension | 3 |  | 0.44 | 374 | 1167 | 168 | 2424 | 0.69 | 0.68 | 0.24 | 0.94 | 0.131 | 2.12 | 0.46 |
|  | 8378 | both | SBP hypertension | 3 |  | 0.46 | 916 | 1842 | 483 | 5137 | 0.65 | 0.74 | 0.33 | 0.91 | 0.167 | 2.48 | 0.47 |
|  | 4245 | boy | DBP hypertension | 3 |  | 0.49 | 559 | 875 | 294 | 2517 | 0.66 | 0.74 | 0.39 | 0.90 | 0.201 | 2.54 | 0.46 |
|  | 4133 | girl | DBP hypertension | 3 |  | 0.47 | 237 | 657 | 305 | 2934 | 0.44 | 0.82 | 0.27 | 0.91 | 0.131 | 2.39 | 0.69 |
|  | 8378 | both | DBP hypertension | 3 |  | 0.47 | 802 | 1821 | 597 | 5158 | 0.57 | 0.74 | 0.31 | 0.90 | 0.167 | 2.20 | 0.58 |
|  | 4245 | boy | hypertension | 3 |  | 0.49 | 587 | 685 | 266 | 2707 | 0.69 | 0.80 | 0.46 | 0.91 | 0.201 | 3.41 | 0.39 |
|  | 4133 | girl | hypertension | 3 |  | 0.44 | 351 | 1156 | 190 | 2436 | 0.65 | 0.68 | 0.23 | 0.93 | 0.131 | 2.02 | 0.52 |
|  | 8378 | both | hypertension | 3 |  | 0.46 | 881 | 1815 | 518 | 5164 | 0.63 | 0.74 | 0.33 | 0.91 | 0.167 | 2.42 | 0.50 |
| Motswagole BS(9), 2011 | 321 | boy | high BP | 3 | - | 0.41 | 26 | 129 | 16 | 150 | 0.62 | 0.54 | 0.17 | 0.90 | 0.131 | 1.34 | 0.71 |
|  | 367 | girl | high BP | 3 |  | 0.41 | 46 | 144 | 29 | 148 | 0.61 | 0.51 | 0.24 | 0.84 | 0.204 | 1.24 | 0.76 |
| Kromeyer-Hauschild K(10), 2013 | 3492 | boy | hypertension | 3 | the narrowest part of the torso | 0.5 | 117 | 254 | 278 | 2843 | 0.30 | 0.92 | 0.32 | 0.91 | 0.113 | 3.61 | 0.77 |
|  | 3321 | girl | hypertension | 3 |  | 0.5 | 73 | 192 | 249 | 2807 | 0.23 | 0.94 | 0.28 | 0.92 | 0.097 | 3.54 | 0.83 |
| Chiolero A(11), 2013 | 5207 | both | elevated BP | 3 | The midpoint of the upper edge of the ilium muscle and the lower edge of the ribs | 0.5 | 137 | 417 | 435 | 4218 | 0.24 | 0.91 | 0.25 | 0.91 | 0.110 | 2.66 | 0.84 |
| Cheah WL(12), 2018 | 1033 | boy | hypertension | 3 | The midpoint of the upper edge of the ilium muscle and the lower edge of the ribs | 0.42 | 165 | 316 | 67 | 485 | 0.71 | 0.61 | 0.34 | 0.88 | 0.225 | 1.80 | 0.48 |
|  |  |  |  |  |  |  |  |  |  |  |  |  |  |  |  |  |  |
|  | 1428 | girl | hypertension | 3 |  | 0.44 | 132 | 498 | 52 | 746 | 0.72 | 0.60 | 0.21 | 0.93 | 0.129 | 1.79 | 0.47 |
| MENG LH(13), 2008 | 4939 | both | 1 RF | 1 | The midpoint of the upper edge of the ilium muscle and the lower edge of the ribs | 0.48 | 1989 | 293 | 732 | 1925 | 0.73 | 0.87 | 0.87 | 0.72 | 0.551 | 5.53 | 0.31 |
|  | 4883 | both | ≥2 RFs | 1 |  | 0.48 | 985 | 1254 | 87 | 2557 | 0.92 | 0.67 | 0.44 | 0.97 | 0.220 | 2.79 | 0.12 |
|  | 4836 | both | 3RFs | 1 |  | 0.48 | 222 | 1996 | 4 | 2614 | 0.98 | 0.57 | 0.10 | 1.00 | 0.047 | 2.27 | 0.03 |
|  | 4875 | both | high BP | 3 |  | 0.48 | 679 | 1551 | 280 | 2365 | 0.71 | 0.60 | 0.30 | 0.89 | 0.197 | 1.79 | 0.48 |
|  | 4846 | both | dyslipidmia | 4 |  | 0.48 | 707 | 1503 | 419 | 2217 | 0.63 | 0.60 | 0.32 | 0.84 | 0.232 | 1.55 | 0.62 |
| Christofaro DGD(14), 2018 | 8295 | both | hypertension | 3 | at the level of the umbilicus | 0.5 | 381 | 1201 | 847 | 5866 | 0.31 | 0.83 | 0.24 | 0.87 | 0.148 | 1.83 | 0.83 |
| MA CW(15), 2016 | 5346 | boy | elevated BP | 3 | The midpoint of the upper edge of the ilium muscle and the lower edge of the ribs | 0.48 | 275 | 1087 | 368 | 3616 | 0.43 | 0.77 | 0.20 | 0.91 | 0.120 | 1.85 | 0.74 |
|  | 4817 | girl | elevated BP | 3 |  | 0.46 | 134 | 612 | 394 | 3677 | 0.25 | 0.86 | 0.18 | 0.90 | 0.110 | 1.78 | 0.87 |
| Beck CC(16), 2011 | 317 | boy | high BP | 3 | The midpoint of the upper edge of the ilium muscle and the lower edge of the ribs | 0.43 | 13 | 99 | 5 | 200 | 0.72 | 0.67 | 0.12 | 0.98 | 0.057 | 2.18 | 0.42 |
|  | 343 | girl | high BP | 3 |  | 0.48 | 4 | 52 | 0 | 287 | 1.00 | 0.85 | 0.07 | 1.00 | 0.012 | 6.52 | 0.00 |
| Wariri O(17), 2018 | 367 | both | elevated BP | 3 | The midpoint of the upper edge of the ilium muscle and the lower edge of the ribs | 0.5 | 26 | 53 | 13 | 275 | 0.67 | 0.84 | 0.33 | 0.95 | 0.106 | 4.13 | 0.40 |
| Mishra PE(18), 2015 | 1913 | both | high SBP (pre-hypertension) | 3 | The midpoint of the upper edge of the ilium muscle and the lower edge of the ribs | 0.5 | 42 | 208 | 111 | 1552 | 0.27 | 0.88 | 0.17 | 0.93 | 0.080 | 2.32 | 0.82 |
|  | 1913 | both | high DBP (pre-hypertension) | 3 |  | 0.5 | 18 | 251 | 33 | 1611 | 0.35 | 0.87 | 0.07 | 0.98 | 0.027 | 2.62 | 0.75 |
| LIU Y(19), 2007 | 962 | both | dyslipidmia | 4 | - | 0.43 | 86 | 628 | 21 | 227 | 0.80 | 0.27 | 0.12 | 0.92 | 0.111 | 1.09 | 0.74 |
| [Zheng W](https://www.ncbi.nlm.nih.gov/pubmed/?term=Zheng%20W%5BAuthor%5D&cauthor=true&cauthor_uid=27129304)(20), 2016 | 399 | boy | dyslipidmia | 4 | 1cm above the umbilicus | 0.473 | 29 | 82 | 20 | 268 | 0.59 | 0.77 | 0.26 | 0.93 | 0.123 | 2.53 | 0.53 |
| Chen G(21), 2019 | 255 | boy | abdominal fat | 5 | around the same anatomical sites | 0.51 | 42 | 23 | 10 | 180 | 0.81 | 0.89 | 0.65 | 0.95 | 0.204 | 7.13 | 0.22 |
|  | 197 | girl | abdominal fat | 5 |  | 0.47 | 27 | 32 | 6 | 132 | 0.82 | 0.80 | 0.46 | 0.96 | 0.168 | 4.19 | 0.23 |
|  |  |  |  |  |  |  |  |  |  |  |  |  |  |  |  |  |  |
| Ejtahed HS(22), 2019 | 7019 | boy | central obesity | 5 | between the uppermost lateral border of right ilium and that of left ilium | 0.5 | 685 | 628 | 52 | 5654 | 0.93 | 0.90 | 0.52 | 0.99 | 0.105 | 9.30 | 0.08 |
|  | 7214 | girl | central obesity | 5 |  | 0.5 | 672 | 781 | 35 | 5726 | 0.95 | 0.88 | 0.46 | 0.99 | 0.098 | 7.92 | 0.06 |
| Dong B(23), 2016 | 60436 | boy | abdominally overweight | 5 | 1cm above the umbilicus | 0.44 | 16224 | 4687 | 1605 | 37920 | 0.91 | 0.89 | 0.78 | 0.96 | 0.295 | 8.27 | 0.10 |
|  | 60590 | girl | abdominally overweight | 5 |  | 0.44 | 15338 | 2948 | 3142 | 39162 | 0.83 | 0.93 | 0.84 | 0.93 | 0.305 | 11.86 | 0.18 |
| Fujita Y(24), 2011 | 226 | boy | abdominal fat | 5 | The midpoint of the upper edge of the ilium muscle and the lower edge of the ribs | 0.519 | 12 | 11 | 0 | 203 | 1.00 | 0.95 | 0.52 | 1.00 | 0.053 | 19.45 | 0.00 |
|  | 196 | girl | abdominal fat | 5 |  | 0.499 | 11 | 9 | 0 | 176 | 1.00 | 0.95 | 0.55 | 1.00 | 0.056 | 20.56 | 0.00 |
| Zhou D(25), 2014 | 8843 | boy | meeting 3 criteria of MetS | 1 | The midpoint of the upper edge of the ilium muscle and the lower edge of the ribs | 0.47 | 288 | 1489 | 48 | 7018 | 0.86 | 0.82 | 0.16 | 0.99 | 0.038 | 4.90 | 0.17 |
|  | 8071 | girl | meeting 3 criteria of MetS | 1 |  | 0.45 | 265 | 1460 | 42 | 6304 | 0.86 | 0.81 | 0.15 | 0.99 | 0.038 | 4.59 | 0.17 |
|  | 8843 | boy | central obesity | 5 |  | 0.47 | 1051 | 611 | 64 | 7117 | 0.94 | 0.92 | 0.63 | 0.99 | 0.126 | 11.92 | 0.06 |
|  | 8071 | girl | central obesity | 5 |  | 0.45 | 979 | 670 | 38 | 6384 | 0.96 | 0.91 | 0.59 | 0.99 | 0.126 | 10.13 | 0.04 |
| Dai YL(26), 2014 | 9771 | boy | ≥2 RFs | 1 | The midpoint of the upper edge of the ilium muscle and the lower edge of the ribs | 0.48 | 227 | 1322 | 262 | 7960 | 0.46 | 0.86 | 0.15 | 0.97 | 0.050 | 3.26 | 0.62 |
|  | 8758 | girl | ≥2 RFs | 1 |  | 6~9 years: 0.48; 10~15 years: 0.46 | 123 | 1274 | 178 | 7183 | 0.41 | 0.85 | 0.09 | 0.98 | 0.034 | 2.71 | 0.70 |
| Matsha TE(27), 2013 | 1272 | both | 2 components of MetS | 1 | at the level of the narrowest part of the torso | 0.465 | 44 | 266 | 61 | 901 | 0.42 | 0.77 | 0.14 | 0.94 | 0.083 | 1.84 | 0.75 |
|  | 496 | boy | 2 components of MetS | 1 |  | 0.455 | 27 | 50 | 36 | 383 | 0.43 | 0.88 | 0.35 | 0.91 | 0.127 | 3.71 | 0.65 |
|  | 776 | girl | 2 components of MetS | 1 |  | 0.465 | 21 | 224 | 21 | 510 | 0.50 | 0.69 | 0.09 | 0.96 | 0.054 | 1.64 | 0.72 |
| Bauer KW(28), 2015 | 6029 | both | ≥1 RF | 1 | above the iliac crest | 0.52 | 1432 | 795 | 1270 | 2532 | 0.53 | 0.76 | 0.64 | 0.67 | 0.448 | 2.22 | 0.62 |
|  | 6049 | both | ≥2 RFs | 1 |  | 0.52 | 726 | 1508 | 331 | 3484 | 0.69 | 0.70 | 0.32 | 0.91 | 0.175 | 2.27 | 0.45 |
|  | 6052 | both | ≥3 RFs | 1 |  | 0.52 | 275 | 1958 | 69 | 3750 | 0.80 | 0.66 | 0.12 | 0.98 | 0.057 | 2.33 | 0.31 |
| Liu XL(29), 2015 | 1601 | boy | hypertriglyceridemic waist phenotype | 1 | The midpoint of the upper edge of the ilium muscle and the lower edge of the ribs | 0.48 | 56 | 185 | 1 | 1359 | 0.98 | 0.88 | 0.23 | 1.00 | 0.036 | 8.20 | 0.02 |
|  | 1535 | girl | hypertriglyceridemic waist phenotype | 1 |  | 0.46 | 43 | 197 | 1 | 1294 | 0.98 | 0.87 | 0.18 | 1.00 | 0.029 | 7.40 | 0.03 |
| Seo JY(30), 2017 | 2935 | both | MetS (CMR_2_) | 1 | The midpoint of the upper edge of the ilium muscle and the lower edge of the ribs | 0.491 | 51 | 340 | 2 | 2542 | 0.96 | 0.88 | 0.13 | 1.00 | 0.018 | 8.16 | 0.04 |
| Aguirre P F(31), 2017 | 186 | boy | meeting 3 criteria of MetS | 1 | at the umbilical level | 0.5 | 10 | 58 | 0 | 118 | 1.00 | 0.67 | 0.15 | 1.00 | 0.054 | 3.03 | 0.00 |
|  | 209 | girl | meeting 3 criteria of MetS | 1 |  | 0.5 | 21 | 58 | 0 | 130 | 1.00 | 0.69 | 0.27 | 1.00 | 0.100 | 3.24 | 0.00 |
|  | 186 | boy | meeting 4 criteria of MetS | 1 |  | 0.5 | 2 | 61 | 0 | 123 | 1.00 | 0.67 | 0.03 | 1.00 | 0.011 | 3.02 | 0.00 |
|  | 209 | girl | meeting 4 criteria of MetS | 1 |  | 0.5 | 4 | 64 | 0 | 141 | 1.00 | 0.69 | 0.06 | 1.00 | 0.019 | 3.20 | 0.00 |
| Adegboye AR(32), 2010 | 1385 | boy | 3 RFs | 1 | The midpoint of the upper edge of the ilium muscle and the lower edge of the ribs | ranged by ages | 111 | 490 | 76 | 708 | 0.59 | 0.59 | 0.18 | 0.90 | 0.135 | 1.45 | 0.69 |
|  | 1452 | girl | 3 RFs | 1 |  | ranged by ages | 175 | 385 | 80 | 812 | 0.69 | 0.68 | 0.31 | 0.91 | 0.176 | 2.13 | 0.46 |
| Ma CM(33), 2016 | 1601 | boy | MetS (CMR_3_) | 1 | The midpoint of the upper edge of the ilium muscle and the lower edge of the ribs | 0.48 | 177 | 60 | 8 | 1356 | 0.96 | 0.96 | 0.75 | 0.99 | 0.116 | 22.58 | 0.05 |
|  | 1535 | girl | MetS (CMR_3_) | 1 |  | 0.46 | 145 | 92 | 7 | 1291 | 0.95 | 0.93 | 0.61 | 0.99 | 0.100 | 14.34 | 0.05 |
| Zhao M(34), 2017 | 3622 | both | ≥ 3 criteria of MerS | 1 | at the high point of the iliac crest | 0.52 | 107 | 556 | 42 | 2917 | 0.72 | 0.84 | 0.16 | 0.99 | 0.041 | 4.49 | 0.34 |
|  | 1868 | boy | ≥ 3 criteria of MerS | 1 |  | 0.48 | 87 | 371 | 14 | 1396 | 0.86 | 0.79 | 0.19 | 0.99 | 0.054 | 4.10 | 0.18 |
|  | 1753 | girl | ≥ 3 criteria of MerS | 1 |  | 0.51 | 39 | 409 | 12 | 1293 | 0.76 | 0.76 | 0.09 | 0.99 | 0.029 | 3.18 | 0.31 |
| Xu T(35), 2017 | 6171 | boy | MetS (CMR_3_) | 1 | at the level of the navel | 0.46 | 250 | 1082 | 74 | 4765 | 0.77 | 0.81 | 0.19 | 0.98 | 0.053 | 4.17 | 0.28 |
|  | 5004 | girl | MetS (CMR_3_) | 1 |  | 0.46 | 74 | 1000 | 30 | 3900 | 0.71 | 0.80 | 0.07 | 0.99 | 0.021 | 3.49 | 0.36 |
| Oliveira RG(36), 2018 | 470 | boy | MetS (CMR_3_) | 1 | The midpoint of the upper edge of the ilium muscle and the lower edge of the ribs | ranged by ages | 15 | 164 | 9 | 282 | 0.63 | 0.63 | 0.08 | 0.97 | 0.051 | 1.70 | 0.59 |
|  | 565 | girl | MetS (CMR_3_) | 1 |  | ranged by ages | 14 | 153 | 6 | 392 | 0.70 | 0.72 | 0.09 | 0.98 | 0.036 | 2.48 | 0.42 |
| LIU Bingyang(37), 2017 | 492 | boy | MetS (CMR_3_) | 1 | The midpoint of the upper edge of the ilium muscle and the lower edge of the ribs | 0.48 | 48 | 69 | 7 | 368 | 0.87 | 0.84 | 0.41 | 0.98 | 0.112 | 5.53 | 0.15 |
|  | 436 | girl | MetS (CMR_3_) | 1 |  | 0.46 | 20 | 76 | 1 | 339 | 0.95 | 0.82 | 0.21 | 1.00 | 0.048 | 5.20 | 0.06 |
| Arsang-Jang S(38), 2019 | 7235 | boy | MetS (CMR_3_) | 1 | The midpoint of the upper edge of the ilium muscle and the lower edge of the ribs | 0.513 | 776 | 1104 | 0 | 5355 | 1.00 | 0.83 | 0.41 | 1.00 | 0.107 | 5.85 | 0.00 |
|  | 7051 | girl | MetS (CMR_3_) | 1 |  | 0.53 | 797 | 650 | 62 | 5542 | 0.93 | 0.90 | 0.55 | 0.99 | 0.122 | 8.84 | 0.08 |
| Vasquez Fabian(39), 2019 | 354 | boy | MetS (CMR_3_) | 1 | at the highest point of the iliac crest around the abdomen | 0.54 | 28 | 24 | 2 | 300 | 0.93 | 0.93 | 0.54 | 0.99 | 0.085 | 12.68 | 0.07 |
|  | 324 | girl | MetS (CMR_3_) | 1 |  | 0.54 | 21 | 80 | 8 | 215 | 0.72 | 0.73 | 0.21 | 0.96 | 0.090 | 2.67 | 0.38 |
| Graves L(40), 2014 | 1368 | boy | ≥3 RFs | 1 | The midpoint of the upper edge of the ilium muscle and the lower edge of the ribs | 0.47 | 54 | 209 | 50 | 1055 | 0.52 | 0.83 | 0.21 | 0.95 | 0.076 | 3.14 | 0.58 |
|  | 1488 | girl | ≥3 RFs | 1 |  | 0.48 | 43 | 487 | 21 | 937 | 0.67 | 0.66 | 0.08 | 0.98 | 0.043 | 1.96 | 0.50 |
| Tompuri TT(41), 2019 | 249 | boy | meeting 3 criteria of MetS; | 1 | The midpoint of the upper edge of the ilium muscle and the lower edge of the ribs | 0.452 | 7 | 62 | 3 | 177 | 0.70 | 0.74 | 0.10 | 0.98 | 0.040 | 2.70 | 0.41 |
|  | 233 | girl | meeting 3 criteria of MetS; | 1 |  | 0.46 | 5 | 39 | 1 | 188 | 0.83 | 0.83 | 0.11 | 0.99 | 0.026 | 4.85 | 0.20 |
| Benmohammed K(42), 2015 | 528 | boy | meeting 3 criteria of MetS; | 1 | The midpoint of the upper edge of the ilium muscle and the lower edge of the ribs | 0.5 | 7 | 73 | 0 | 448 | 1.00 | 0.86 | 0.09 | 1.00 | 0.013 | 7.14 | 0.00 |
|  | 560 | girl | meeting 3 criteria of MetS; | 1 |  | 0.55 | 3 | 61 | 0 | 496 | 1.00 | 0.89 | 0.04 | 1.00 | 0.005 | 9.09 | 0.00 |
| Zhang Y(43), 2019 | 683 | both | MetS; | 1 | 1cm above the umbilicus | - | 31 | 91 | 3 | 558 | 0.91 | 0.86 | 0.25 | 0.99 | 0.050 | 6.50 | 0.10 |
| Yuan Y(44), 2020 | 683 | both | FBG; | 2 | 1cm above the umbilicus | - | 38 | 128 | 56 | 461 | 0.40 | 0.78 | 0.23 | 0.89 | 0.138 | 1.86 | 0.76 |
| Wang Y(45),2020 | 683 | both | Hypertension; | 3 | 1cm above the umbilicus | - | 41 | 158 | 39 | 445 | 0.51 | 0.74 | 0.21 | 0.92 | 0.117 | 1.96 | 0.66 |
| Tee JYH(46),2020 | 513 | both | hypertention( 95th percentiles) | 3 | - | 0.44 | 55 | 164 | 6 | 288 | 0.90 | 0.64 | 0.25 | 0.98 | 0.119 | 2.49 | 0.15 |
|  | 211 | boy | hypertention( 95th percentiles) | 3 |  | 0.52 | 17 | 23 | 9 | 162 | 0.65 | 0.88 | 0.43 | 0.95 | 0.123 | 5.26 | 0.40 |
|  | 302 | girl | hypertention( 95th percentiles) | 3 |  | 0.45 | 33 | 91 | 2 | 176 | 0.94 | 0.66 | 0.27 | 0.99 | 0.116 | 2.77 | 0.09 |
|  | 513 | both | hypertention( 90th percentiles) | 3 |  | 0.44 | 118 | 101 | 41 | 253 | 0.74 | 0.71 | 0.54 | 0.86 | 0.310 | 2.60 | 0.36 |
|  | 211 | boy | hypertention( 90th percentiles) | 3 |  | 0.46 | 45 | 29 | 19 | 118 | 0.70 | 0.80 | 0.61 | 0.86 | 0.303 | 3.56 | 0.37 |
|  | 302 | girl | hypertention( 90th percentiles) | 3 |  | 0.45 | 68 | 56 | 27 | 151 | 0.72 | 0.73 | 0.55 | 0.85 | 0.315 | 2.65 | 0.39 |
| Vaquero-Álvarez M(47),2020 | 265 | both | hypertention( 95th percentiles) | 3 | The midpoint of the upper edge of the ilium muscle and the lower edge of the ribs | 0.46 | 13 | 87 | 5 | 159 | 0.72 | 0.65 | 0.13 | 0.97 | 0.068 | 2.04 | 0.43 |
| Silva KC(48),2020 | 548 | both | CMR_3_ | 1 |  | - | 13 | 9 | 0 | 526 | 1.00 | 0.98 | 0.59 | 1.00 | 0.024 | 59.44 | 0.00 |
|  | 238 | boy | CMR_3_ | 1 |  | 0.55 | 9 | 7 | 0 | 222 | 1.00 | 0.97 | 0.56 | 1.00 | 0.038 | 32.71 | 0.00 |
|  | 310 | girl | CMR_3_ | 1 |  | 0.55 | 4 | 2 | 0 | 304 | 1.00 | 0.99 | 0.67 | 1.00 | 0.013 | 153.00 | 0.00 |
| Li Y(49),2020 | 15698 | both | dyslipidaemia | 4 | - | - | 1705 | 2738 | 2580 | 8675 | 0.40 | 0.76 | 0.38 | 0.77 | 0.273 | 1.66 | 0.79 |
|  | 8004 | boy | dyslipidaemia | 4 |  | - | 831 | 1151 | 1315 | 4707 | 0.39 | 0.80 | 0.42 | 0.78 | 0.268 | 1.97 | 0.76 |
|  | 7694 | girl | dyslipidaemia | 4 |  | - | 874 | 1587 | 1265 | 3968 | 0.41 | 0.71 | 0.36 | 0.76 | 0.278 | 1.43 | 0.83 |
|  | 15698 | both | hypertension | 3 |  | - | 770 | 3488 | 888 | 10552 | 0.46 | 0.75 | 0.18 | 0.92 | 0.106 | 1.87 | 0.71 |
|  | 8004 | boy | hypertension | 3 |  | - | 507 | 2087 | 374 | 5036 | 0.58 | 0.71 | 0.20 | 0.93 | 0.110 | 1.96 | 0.60 |
|  | 7694 | girl | hypertension | 3 |  | - | 263 | 1401 | 514 | 5516 | 0.34 | 0.80 | 0.16 | 0.91 | 0.101 | 1.67 | 0.83 |
|  | 15698 | both | CMR_3_ | 1 |  | - | 472 | 3557 | 422 | 11247 | 0.53 | 0.76 | 0.12 | 0.96 | 0.057 | 2.20 | 0.62 |
|  | 8004 | boy | CMR_3_ | 1 |  | - | 271 | 1726 | 185 | 5822 | 0.59 | 0.77 | 0.14 | 0.97 | 0.057 | 2.60 | 0.53 |
|  | 7694 | girl | CMR_3_ | 1 |  | - | 201 | 1831 | 237 | 5425 | 0.46 | 0.75 | 0.10 | 0.96 | 0.057 | 1.82 | 0.72 |
| Mai TMT(50),2020 | 10936 | both | elevated BP | 3 | The midpoint of the upper edge of the ilium muscle and the lower edge of the ribs | 0.48 | 1574 | 3006 | 1324 | 5032 | 0.54 | 0.63 | 0.34 | 0.79 | 0.265 | 1.45 | 0.73 |
|  | 5541 | boy | elevated BP | 3 |  | - | 1015 | 1449 | 702 | 2375 | 0.59 | 0.62 | 0.41 | 0.77 | 0.310 | 1.56 | 0.66 |
|  | 5408 | girl | elevated BP | 3 |  | - | 656 | 1660 | 512 | 2580 | 0.56 | 0.61 | 0.28 | 0.83 | 0.216 | 1.43 | 0.72 |
|  | 1009 | both | dyslipidaemia | 4 |  | 0.47 | 196 | 213 | 152 | 448 | 0.56 | 0.68 | 0.48 | 0.75 | 0.345 | 1.75 | 0.64 |
|  | 486 | boy | dyslipidaemia | 4 |  | 0.44 | 126 | 176 | 38 | 146 | 0.77 | 0.45 | 0.42 | 0.79 | 0.337 | 1.41 | 0.51 |
|  | 523 | girl | dyslipidaemia | 4 |  | 0.47 | 88 | 67 | 97 | 271 | 0.48 | 0.80 | 0.57 | 0.74 | 0.354 | 2.40 | 0.65 |
|  | 1009 | both | CMR_3_ | 1 |  | 0.5 | 79 | 190 | 45 | 695 | 0.64 | 0.79 | 0.29 | 0.94 | 0.123 | 2.97 | 0.46 |
|  | 486 | boy | CMR_3_ | 1 |  | 0.5 | 43 | 120 | 17 | 306 | 0.72 | 0.72 | 0.26 | 0.95 | 0.123 | 2.54 | 0.39 |
|  | 523 | girl | CMR_3_ | 1 |  | 0.47 | 44 | 124 | 19 | 336 | 0.70 | 0.73 | 0.26 | 0.95 | 0.120 | 2.59 | 0.41 |
| Yazdi M(51),2020 | 14008 | both | elevated BP | 3 | The midpoint of the upper edge of the ilium muscle and the lower edge of the ribs | 0.432 | 1727 | 7095 | 688 | 4498 | 0.72 | 0.39 | 0.20 | 0.87 | 0.172 | 1.17 | 0.73 |
|  | 7091 | boy | elevated BP | 3 |  | 0.421 | 998 | 4131 | 225 | 1737 | 0.82 | 0.30 | 0.19 | 0.89 | 0.172 | 1.16 | 0.62 |
|  | 6917 | girl | elevated BP | 3 |  | 0.432 | 840 | 3527 | 323 | 2227 | 0.72 | 0.39 | 0.19 | 0.87 | 0.168 | 1.18 | 0.72 |
|  | 14008 | both | hypertention( 95^th^ percentiles) | 3 |  | 0.475 | 664 | 3721 | 859 | 8764 | 0.44 | 0.70 | 0.15 | 0.91 | 0.109 | 1.46 | 0.80 |
|  | 7091 | boy | hypertention( 95^th^ percentiles) | 3 |  | 0.469 | 365 | 2167 | 372 | 4187 | 0.50 | 0.66 | 0.14 | 0.92 | 0.104 | 1.45 | 0.77 |
|  | 6917 | girl | hypertention( 95^th^ percentiles) | 3 |  | 0.477 | 326 | 1773 | 456 | 4362 | 0.42 | 0.71 | 0.16 | 0.91 | 0.113 | 1.44 | 0.82 |
| Kilinc A(52),2019 | 2718 | both | abnormality obesity | 5 | - | 0.4741 | 645 | 451 | 73 | 1549 | 0.90 | 0.77 | 0.59 | 0.95 | 0.264 | 3.98 | 0.13 |
|  | 1467 | boy | abnormality obesity | 5 |  | 0.4762 | 443 | 208 | 35 | 781 | 0.93 | 0.79 | 0.68 | 0.96 | 0.326 | 4.41 | 0.09 |
|  | 1251 | girl | abnormality obesity | 5 |  | 0.4697 | 238 | 249 | 26 | 738 | 0.90 | 0.75 | 0.49 | 0.97 | 0.211 | 3.57 | 0.13 |
| Arellano‐Ruiz P(53),2020 | 848 | both | HDL-C | 4 | - | 0.52 | 52 | 151 | 48 | 597 | 0.52 | 0.80 | 0.26 | 0.93 | 0.118 | 2.58 | 0.60 |
|  | 848 | both | TG | 4 |  | 0.52 | 61 | 142 | 58 | 587 | 0.51 | 0.81 | 0.30 | 0.91 | 0.140 | 2.63 | 0.61 |
|  | 848 | both | elevated BP(95^th^ percentiles) | 3 |  | 0.57 | 9 | 67 | 18 | 754 | 0.33 | 0.92 | 0.12 | 0.98 | 0.032 | 4.08 | 0.73 |
|  | 848 | both | MetS (CMR_3_) | 1 |  | 0.51 | 164 | 78 | 114 | 492 | 0.59 | 0.86 | 0.68 | 0.81 | 0.328 | 4.31 | 0.48 |

^a^ 1. Clustering of cardiometabolic risk factors; 2. Elevated fasting blood glucose; 3. Elevated blood pressure; 4. Dyslipidaemia; 5. Central obesity.

MetS, metabolic syndrome; CMR: cardiometabolic risk factor; CMR_1_: presenting with at least one of CMRs; CMR_2_: presenting with at least two CMRs; CMR_3_: presenting with at least three CMRs; RF, risk factor; FBG: fasting blood glucose; BP, blood pressure; SBP, systolic blood pressure; DBP, diastolic blood pressure; TC, total cholesterol; TG, triglyceride; HDL-C, High-density leptin cholesterol; LDL-C, Low-density leptin cholesterol.

PPV: positive predictive value; NPV: negative predictive value; PLR: positive likelihood ratio; NLR: negative likelihood ratio.

The sensitivity, specificity, PPV, NPV, PLR, NLR and prevalence of outcomes were imputed based on fourfold tables extracted from original papers.

**References:**

1. Dou Y, Jiang Y, Yan Y, Chen H, Zhang Y, Chen X, et al. Waist-to-height ratio as a screening tool for cardiometabolic risk in children and adolescents: a nationwide cross-sectional study in China. BMJ Open. 2020;10(6):e037040.

2. Nan Zh, Cui L, Cui MH, Xu MH, Jin YH, Fang JN. Relationships of different types of obesity with metabolic syndrome and its components among Han-Chinese adolescents in Yanbian area. Chinese Journal of School Health. 2013;34(4):457-9.

3. HOU YP, YANG L, XI B. Comparison of the performance of waist circumference， waist-height ratio， and body mass index in predicting metabolic disorders among children and adolescents. Chinese Journal of Child Health Care. 2018;26(3):239-42,57.

4. Perona JS, Schmidt-RioValle J, Rueda-Medina B, Correa-Rodriguez M, Gonzalez-Jimenez E. Waist circumference shows the highest predictive value for metabolic syndrome, and waist-to-hip ratio for its components, in Spanish adolescents. Nutr Res. 2017;45:38-45.

5. Quadros TM, Gordia AP, Mota J, Silva LR. Utility of body mass index, waist circumference and waist-to-height ratio as screening tools for hyperglycemia in young people. Arch Endocrinol Metab. 2016;60(6):526-31.

6. Lopez-Gonzalez D, Miranda-Lora A, Klunder-Klunder M, Queipo-Garcia G, Bustos-Esquivel M, Paez-Villa M, et al. DIAGNOSTIC PERFORMANCE OF WAIST CIRCUMFERENCE MEASUREMENTS FOR PREDICTING CARDIOMETABOLIC RISK IN MEXICAN CHILDREN. Endocr Pract. 2016;22(10):1170-6.

7. Kruger HS, Faber M, Schutte AE, Ellis SM. A proposed cutoff point of waist-to-height ratio for metabolic risk in African township adolescents. Nutrition. 2013;29(3):502-7.

8. Xue J. The predictive effect of obesity-related indicators and blood pressure to height ratio on hypertension among urban school-age children: Shandong University; 2014.

9. Motswagole BS, Kruger HS, Faber M, van Rooyen JM, de Ridder JH. The sensitivity of waist-to-height ratio in identifying children with high blood pressure. Cardiovasc J Afr. 2011;22(4):208-11.

10. Kromeyer-Hauschild K, Neuhauser H, Schaffrath Rosario A, Schienkiewitz A. Abdominal obesity in German adolescents defined by waist-to-height ratio and its association to elevated blood pressure: the KiGGS study. Obes Facts. 2013;6(2):165-75.

11. Chiolero A, Paradis G, Maximova K, Burnier M, Bovet P. No use for waist-for-height ratio in addition to body mass index to identify children with elevated blood pressure. Blood Press. 2013;22(1):17-20.

12. Cheah WL, Chang CT, Hazmi H, Kho GWF. Using Anthropometric Indicator to Identify Hypertension in Adolescents: A Study in Sarawak, Malaysia. Int J Hypertens. 2018;2018:6736251.

13. Meng Lh, Mi J. The validation of the classification criterion of waist and waist-to-height ratio for cardiometabolic risk factors in Chinese school-age children. Chinese Journal of Evidence Based Pediatrics. 2008;3(5):324-32.

14. Christofaro DGD, Farah BQ, Vanderlei LCM, Delfino LD, Tebar WR, Barros MVG, et al. Analysis of different anthropometric indicators in the detection of high blood pressure in school adolescents: a cross-sectional study with 8295 adolescents. Braz J Phys Ther. 2018;22(1):49-54.

15. Ma CW, Liang YJ, Xi B. Comparison of the performance of waist circumference and waist-height ratio in predicting elevated blood pressure among children and adolescents. Chinese Journal of School Health. 2016;37(10):1445-8.

16. Beck CC, Lopes Ada S, Pitanga FJ. Anthropometric indicators as predictors of high blood pressure in adolescents. Arq Bras Cardiol. 2011;96(2):126-33.

17. Wariri O, Jalo I, Bode-Thomas F. Discriminative ability of adiposity measures for elevated blood pressure among adolescents in a resource-constrained setting in northeast Nigeria: a cross-sectional analysis. BMC Obes. 2018;5:35.

18. Mishra PE, Shastri L, Thomas T, Duggan C, Bosch R, McDonald CM, et al. Waist-to-Height Ratio as an Indicator of High Blood Pressure in Urban Indian School Children. Indian Pediatr. 2015;52(9):773-8.

19. Liu Y, Mi J, Han W, Jin Hf, Du Jb. Analyze the indices of the screening test of hyperlipidemia by Logistic regression analysis and ROC study in children. BASIC & CLINICAL MEDICINE. 2007;27(2):152-6.

20. Zheng W, Zhao A, Xue Y, Zheng Y, Chen Y, Mu Z, et al. Gender and urban-rural difference in anthropometric indices predicting dyslipidemia in Chinese primary school children: a cross-sectional study. Lipids Health Dis. 2016;15:87.

21. Chen G, Yan H, Hao Y, Shrestha S, Wang J, Li Y, et al. Comparison of various anthropometric indices in predicting abdominal obesity in Chinese children: a cross-sectional study. BMC Pediatr. 2019;19(1):127.

22. Ejtahed HS, Kelishadi R, Qorbani M, Motlagh ME, Hasani-Ranjbar S, Angoorani P, et al. Utility of waist circumference-to-height ratio as a screening tool for generalized and central obesity among Iranian children and adolescents: The CASPIAN-V study. Pediatr Diabetes. 2019;20(5):530-7.

23. Dong B, Wang Z, Arnold LW, Song Y, Wang HJ, Ma J. Simplifying the screening of abdominal adiposity in Chinese children with waist-to-height ratio. Am J Hum Biol. 2016;28(6):945-9.

24. Fujita Y, Kouda K, Nakamura H, Iki M. Cut-off values of body mass index, waist circumference, and waist-to-height ratio to identify excess abdominal fat: population-based screening of Japanese school children. J Epidemiol. 2011;21(3):191-6.

25. Zhou D, Yang M, Yuan ZP, Zhang DD, Liang L, Wang CL, et al. Waist-to-Height Ratio: a simple, effective and practical screening tool for childhood obesity and metabolic syndrome. Prev Med. 2014;67:35-40.

26. Dai Y, Fu J, Liang L, Gong C, Xiong F, Liu G, et al. [A proposal for the cutoff point of waist-to-height for the diagnosis of metabolic syndrome in children and adolescents in six areas of China]. Zhonghua Liu Xing Bing Xue Za Zhi. 2014;35(8):882-5.

27. Matsha TE, Kengne AP, Yako YY, Hon GM, Hassan MS, Erasmus RT. Optimal waist-to-height ratio values for cardiometabolic risk screening in an ethnically diverse sample of South African urban and rural school boys and girls. PLoS One. 2013;8(8):e71133.

28. Bauer KW, Marcus MD, El ghormli L, Ogden CL, Foster GD. Cardio-metabolic risk screening among adolescents: understanding the utility of body mass index, waist circumference and waist to height ratio. Pediatr Obes. 2015;10(5):329-37.

29. Liu XL, Yin FZ, Ma CP, Gao GQ, Ma CM, Wang R, et al. Waist-to-height ratio as a screening measure for identifying adolescents with hypertriglyceridemic waist phenotype. J Pediatr Endocrinol Metab. 2015;28(9-10):1079-83.

30. Seo JY, Kim JH. Validation of surrogate markers for metabolic syndrome and cardiometabolic risk factor clustering in children and adolescents: A nationwide population-based study. PLoS One. 2017;12(10):e0186050.

31. Aguirre PF, Coca A, Aguirre MF, Celis G. Waist-to-height ratio and sedentary lifestyle as predictors of metabolic syndrome in children in Ecuador. Hipertens Riesgo Vasc. 2017.

32. Adegboye AR, Andersen LB, Froberg K, Sardinha LB, Heitmann BL. Linking definition of childhood and adolescent obesity to current health outcomes. Int J Pediatr Obes. 2010;5(2):130-42.

33. Ma CM, Yin FZ, Liu XL, Wang R, Lou DH, Lu Q. How to Simplify the Diagnostic Criteria of Metabolic Syndrome in Adolescents. Pediatr Neonatol. 2017;58(2):178-84.

34. Zhao M, Bovet P, Ma C, Xi B. Performance of different adiposity measures for predicting cardiovascular risk in adolescents. Sci Rep. 2017;7:43686.

35. Xu T, Liu J, Liu J, Zhu G, Han S. Relation between metabolic syndrome and body compositions among Chinese adolescents and adults from a large-scale population survey. BMC Public Health. 2017;17(1):337.

36. Oliveira RG, Guedes DP. Performance of anthropometric indicators as predictors of metabolic syndrome in Brazilian adolescents. BMC Pediatr. 2018;18(1):33.

37. Liu BY, Jiang Rh, Li P, Liu C, Li L. Cutoff Waist-to-height and Waist-to-hip Ratios for Metabolic Syndrome in Chinese Children and Adolescents. Journal of China Medical University. 2017;46(5):434-8,43.

38. Arsang-Jang S, Kelishadi R, Esmail Motlagh M, Heshmat R, Mansourian M. Temporal Trend of Non-Invasive Method Capacity for Early Detection of Metabolic Syndrome in Children and Adolescents: A Bayesian Multilevel Analysis of Pseudo-Panel Data. Ann Nutr Metab. 2019;75(1):55-65.

39. Vasquez F, Correa-Burrows P, Blanco E, Gahagan S, Burrows R. A waist-to-height ratio of 0.54 is a good predictor of metabolic syndrome in 16-year-old male and female adolescents. Pediatr Res. 2019;85(3):269-74.

40. Graves L, Garnett SP, Cowell CT, Baur LA, Ness A, Sattar N, et al. Waist-to-height ratio and cardiometabolic risk factors in adolescence: findings from a prospective birth cohort. Pediatr Obes. 2014;9(5):327-38.

41. Tompuri TT, Jaaskelainen J, Lindi V, Laaksonen DE, Eloranta AM, Viitasalo A, et al. Adiposity Criteria in Assessing Increased Cardiometabolic Risk in Prepubertal Children. Front Endocrinol (Lausanne). 2019;10:410.

42. Benmohammed K, Valensi P, Benlatreche M, Nguyen MT, Benmohammed F, Paries J, et al. Anthropometric markers for detection of the metabolic syndrome in adolescents. Diabetes Metab. 2015;41(2):138-44.

43. Zhang Y, Hu J, Li Z, Li T, Chen M, Wu L, et al. A Novel Indicator Of Lipid Accumulation Product Associated With Metabolic Syndrome In Chinese Children And Adolescents. Diabetes Metab Syndr Obes. 2019;12:2075-83.

44. Yuan Y, Xie H, Sun L, Wang B, Zhang L, Han H, et al. A Novel Indicator of Children's Lipid Accumulation Product Associated with Impaired Fasting Glucose in Chinese Children and Adolescents. Diabetes Metab Syndr Obes. 2020;13:1653-60.

45. Wang Y, Liu W, Sun L, Zhang Y, Wang B, Yuan Y, et al. A novel indicator, childhood lipid accumulation product, is associated with hypertension in Chinese children and adolescents. Hypertens Res. 2020;43(4):305-12.

46. Tee JYH, Gan WY, Lim PY. Comparisons of body mass index, waist circumference, waist-to-height ratio and a body shape index (ABSI) in predicting high blood pressure among Malaysian adolescents: a cross-sectional study. BMJ Open. 2020;10(1):e032874.

47. Vaquero-Álvarez M, Molina-Luque R, Fonseca-Pozo FJ, Molina-Recio G, López-Miranda J, Romero-Saldaña M. Diagnostic Precision of Anthropometric Variables for the Detection of Hypertension in Children and Adolescents. Int J Environ Res Public Health. 2020;17(12).

48. Cristine Silva K, Santana Paiva N, Rocha de Faria F, Franceschini S, Eloiza Piore S. Predictive Ability of Seven Anthropometric Indices for Cardiovascular Risk Markers and Metabolic Syndrome in Adolescents. J Adolesc Health. 2020;66(4):491-8.

49. Li Y, Zou Z, Luo J, Ma J, Ma Y, Jing J, et al. The predictive value of anthropometric indices for cardiometabolic risk factors in Chinese children and adolescents: A national multicenter school-based study. PLoS One. 2020;15(1):e0227954.

50. Mai TMT, Gallegos D, Jones L, Tran QC, Tran TMH, van der Pols JC. The utility of anthopometric indicators to identify cardiovascular risk factors in Vietnamese children. Br J Nutr. 2020;123(9):1043-55.

51. Yazdi M, Assadi F, Qorbani M, Daniali SS, Heshmat R, Esmaeil Motlagh M, et al. Validity of anthropometric indices in predicting high blood pressure risk factors in Iranian children and adolescents: CASPIAN-V study. J Clin Hypertens (Greenwich). 2020;22(6):1009-17.

52. Kilinc A, Col N, Demircioglu-Kilic B, Aydin N, Balat A, Keskin M. Waist to height ratio as a screening tool for identifying childhood obesity and associated factors. Pak J Med Sci. 2019;35(6):1652-8.

53. Arellano-Ruiz P, García-Hermoso A, García-Prieto JC, Sánchez-López M, Vizcaíno VM, Solera-Martínez M. Predictive Ability of Waist Circumference and Waist-to-Height Ratio for Cardiometabolic Risk Screening among Spanish Children. Nutrients. 2020;12(2).
