# Supplementary material for: Performance of waist-to-height ratio as a screening tool for identifying cardiometabolic risk in children: a meta-analysis
Source: Diabetol Metab Syndr. 2021 Jun 14;13:66. doi: 10.1186/s13098-021-00688-7 (PMC8201900; doi:10.1186/s13098-021-00688-7)
Supplement: Supplementary file 3 — Additional file 3: Table S2. Evaluation of the risk of bias and applicability of included studies by QUADAS-2. [file 13098_2021_688_MOESM3_ESM.docx]

**Table S2. Evaluation of the risk of bias and applicability of included studies by QUADAS-2**

|  | DOMAIN 1: PATIENT SELECTION | |  | DOMAIN 2: INDEX TEST(S) | |  | DOMAIN 3: REFERENCE STANDARD | |  | DOMAIN 4: FLOW AND TIMING |
| --- | --- | --- | --- | --- | --- | --- | --- | --- | --- | --- |
| Authors, year | Could the selection of patients have introduced bias? | Is there concern that the included patients do not match the review question? |  | Could the conduct or interpretation of the index test have introduced bias? | Is there concern that the index test, its conduct, or interpretation differ from the review question? |  | Could the reference standard, its conduct, or its interpretation have introduced bias? | Is there concern that the target condition as defined by the reference standard does not match the review question? |  | Could the patient flow have introduced bias? RISK: LOW /HIGH/UNCLEAR |
| Dou YL(1), 2019 | low | low |  | low | low |  | low | low |  | low |
| NAN ZH(2), 2013 | low | low |  | low | low |  | low | low |  | low |
| HOU YP(3), 2018 | low | low |  | low | low |  | high | low |  | high |
| Perona JS(4), 2017 | low | low |  | low | low |  | low | low |  | low |
| Quadros TM(5), 2016 | low | low |  | low | low |  | low | low |  | low |
| López-González D(6), 2016 | low | low |  | low | low |  | low | low |  | low |
| Kruger HS(7), 2012 | high | low |  | low | low |  | low | low |  | low |
| Xue J(8), 2014 | low | low |  | low | low |  | low | low |  | low |
| Motswagole BS(9), 2011 | low | low |  | low | low |  | low | low |  | high |
| Kromeyer-Hauschild K(10), 2013 | low | low |  | low | low |  | low | low |  | low |
| Chiolero A(11), 2013 | low | low |  | low | low |  | low | low |  | low |
| Cheah WL(12), 2018 | low | low |  | low | low |  | low | low |  | low |
| MENG LH(13), 2008 | high | high |  | low | low |  | low | low |  | low |
| Christofaro DGD(14), 2018 | low | low |  | low | low |  | low | low |  | low |
| MA CW(15), 2016 | low | low |  | low | low |  | low | low |  | low |
| Beck CC(16), 2010 | low | low |  | low | low |  | low | low |  | low |
| Wariri O(17), 2018 | low | low |  | low | low |  | low | low |  | low |
| Mishra PE(18), 2015 | low | low |  | low | low |  | low | low |  | low |
| LIU Y(19), 2007 | low | low |  | low | low |  | low | low |  | low |
| Zheng W(20), 2016 | low | low |  | low | low |  | low | low |  | low |
| Chen G(21), 2019 | high | low |  | low | low |  | low | low |  | low |
| Ejtahed HS(22), 2019 | low | low |  | low | low |  | low | low |  | unclear |
| Dong B(23), 2016 | low | low |  | low | low |  | low | low |  | low |
| Fujita Y(24), 2011 | low | low |  | low | low |  | low | low |  | low |
| Zhou D(25), 2014 | low | low |  | low | low |  | low | low |  | low |
| Dai YL(26), 2014 | low | low |  | low | low |  | low | low |  | low |
| Matsha TE(27), 2013 | low | low |  | low | low |  | high | high |  | low |
| Bauer KW(28), 2015 | low | low |  | low | low |  | low | low |  | low |
| Liu XL(29), 2015 | low | low |  | low | low |  | low | low |  | low |
| Seo JY(30), 2017 | low | low |  | low | low |  | low | low |  | low |
| Aguirre PF(31), 2017 | low | low |  | low | low |  | low | low |  | low |
| Adegboye AR(32), 2010 | low | low |  | low | low |  | low | low |  | low |
| Ma CM(33), 2017 | low | low |  | low | low |  | low | low |  | low |
| Zhao M(34), 2017 | low | low |  | low | low |  | low | low |  | low |
| Xu T(35), 2017 | low | low |  | low | low |  | low | low |  | low |
| Oliveira RG(36), 2018 | low | low |  | low | low |  | low | low |  | low |
| LIU BY(37), 2017 | low | low |  | low | low |  | low | low |  | low |
| Arsang-Jang S(38), 2019 | low | low |  | low | low |  | low | low |  | low |
| Vasquez F(39), 2019 | low | low |  | low | low |  | low | low |  | low |
| Graves L(40), 2013 | low | low |  | low | low |  | low | low |  | low |
| Tompuri TT(41), 2019 | low | low |  | low | low |  | low | low |  | low |
| Benmohammed K(42), 2015 | low | low |  | low | low |  | low | low |  | low |
| Yuan Y(43), 2020 | low | low |  | low | low |  | low | low |  | low |
| Zhang Y(44), 2019 | low | low |  | low | low |  | low | low |  | low |
| Wang Y(45), 2020 | low | low |  | low | low |  | low | low |  | low |
| Tee JYH(46), 2020 | low | low |  | low | low |  | low | low |  | low |
| Vaquero-Álvarez M(47), 2020 | low | low |  | low | low |  | low | low |  | low |
| Cristine Silva K(48), 2020 | low | low |  | low | low |  | low | low |  | low |
| Arellano-Ruiz P(49), 2020 | low | low |  | low | low |  | low | low |  | low |
| Li Y(50), 2020 | low | low |  | low | low |  | low | low |  | low |
| Mai T.M.T(51), 2020 | low | low |  | low | low |  | low | low |  | low |
| Yazdi M(52), 2020 | low | low |  | low | low |  | low | low |  | low |
| Kilinc A(53), 2019 | low | low |  | low | low |  | low | low |  | low |

**References**

1. Dou Y, Jiang Y, Yan Y, Chen H, Zhang Y, Chen X, et al. Waist-to-height ratio as a screening tool for cardiometabolic risk in children and adolescents: a nationwide cross-sectional study in China. BMJ Open. 2020;10(6):e037040.

2. Nan Zh, Cui L, Cui MH, Xu MH, Jin YH, Fang JN. Relationships of different types of obesity with metabolic syndrome and its components among Han-Chinese adolescents in Yanbian area. Chinese Journal of School Health. 2013;34(4):457-9.

3. HOU YP, YANG L, XI B. Comparison of the performance of waist circumference， waist-height ratio， and body mass index in predicting metabolic disorders among children and adolescents. Chinese Journal of Child Health Care. 2018;26(3):239-42,57.

4. Perona JS, Schmidt-RioValle J, Rueda-Medina B, Correa-Rodriguez M, Gonzalez-Jimenez E. Waist circumference shows the highest predictive value for metabolic syndrome, and waist-to-hip ratio for its components, in Spanish adolescents. Nutr Res. 2017;45:38-45.

5. Quadros TM, Gordia AP, Mota J, Silva LR. Utility of body mass index, waist circumference and waist-to-height ratio as screening tools for hyperglycemia in young people. Arch Endocrinol Metab. 2016;60(6):526-31.

6. Lopez-Gonzalez D, Miranda-Lora A, Klunder-Klunder M, Queipo-Garcia G, Bustos-Esquivel M, Paez-Villa M, et al. DIAGNOSTIC PERFORMANCE OF WAIST CIRCUMFERENCE MEASUREMENTS FOR PREDICTING CARDIOMETABOLIC RISK IN MEXICAN CHILDREN. Endocr Pract. 2016;22(10):1170-6.

7. Kruger HS, Faber M, Schutte AE, Ellis SM. A proposed cutoff point of waist-to-height ratio for metabolic risk in African township adolescents. Nutrition. 2013;29(3):502-7.

8. Xue J. The predictive effect of obesity-related indicators and blood pressure to height ratio on hypertension among urban school-age children: Shandong University; 2014.

9. Motswagole BS, Kruger HS, Faber M, van Rooyen JM, de Ridder JH. The sensitivity of waist-to-height ratio in identifying children with high blood pressure. Cardiovasc J Afr. 2011;22(4):208-11.

10. Kromeyer-Hauschild K, Neuhauser H, Schaffrath Rosario A, Schienkiewitz A. Abdominal obesity in German adolescents defined by waist-to-height ratio and its association to elevated blood pressure: the KiGGS study. Obes Facts. 2013;6(2):165-75.

11. Chiolero A, Paradis G, Maximova K, Burnier M, Bovet P. No use for waist-for-height ratio in addition to body mass index to identify children with elevated blood pressure. Blood Press. 2013;22(1):17-20.

12. Cheah WL, Chang CT, Hazmi H, Kho GWF. Using Anthropometric Indicator to Identify Hypertension in Adolescents: A Study in Sarawak, Malaysia. Int J Hypertens. 2018;2018:6736251.

13. Meng Lh, Mi J. The validation of the classification criterion of waist and waist-to-height ratio for cardiometabolic risk factors in Chinese school-age children. Chinese Journal of Evidence Based Pediatrics. 2008;3(5):324-32.

14. Christofaro DGD, Farah BQ, Vanderlei LCM, Delfino LD, Tebar WR, Barros MVG, et al. Analysis of different anthropometric indicators in the detection of high blood pressure in school adolescents: a cross-sectional study with 8295 adolescents. Braz J Phys Ther. 2018;22(1):49-54.

15. Ma CW, Liang YJ, Xi B. Comparison of the performance of waist circumference and waist-height ratio in predicting elevated blood pressure among children and adolescents. Chinese Journal of School Health. 2016;37(10):1445-8.

16. Beck CC, Lopes Ada S, Pitanga FJ. Anthropometric indicators as predictors of high blood pressure in adolescents. Arq Bras Cardiol. 2011;96(2):126-33.

17. Wariri O, Jalo I, Bode-Thomas F. Discriminative ability of adiposity measures for elevated blood pressure among adolescents in a resource-constrained setting in northeast Nigeria: a cross-sectional analysis. BMC Obes. 2018;5:35.

18. Mishra PE, Shastri L, Thomas T, Duggan C, Bosch R, McDonald CM, et al. Waist-to-Height Ratio as an Indicator of High Blood Pressure in Urban Indian School Children. Indian Pediatr. 2015;52(9):773-8.

19. Liu Y, Mi J, Han W, Jin Hf, Du Jb. Analyze the indices of the screening test of hyperlipidemia by Logistic regression analysis and ROC study in children. BASIC & CLINICAL MEDICINE. 2007;27(2):152-6.

20. Zheng W, Zhao A, Xue Y, Zheng Y, Chen Y, Mu Z, et al. Gender and urban-rural difference in anthropometric indices predicting dyslipidemia in Chinese primary school children: a cross-sectional study. Lipids Health Dis. 2016;15:87.

21. Chen G, Yan H, Hao Y, Shrestha S, Wang J, Li Y, et al. Comparison of various anthropometric indices in predicting abdominal obesity in Chinese children: a cross-sectional study. BMC Pediatr. 2019;19(1):127.

22. Ejtahed HS, Kelishadi R, Qorbani M, Motlagh ME, Hasani-Ranjbar S, Angoorani P, et al. Utility of waist circumference-to-height ratio as a screening tool for generalized and central obesity among Iranian children and adolescents: The CASPIAN-V study. Pediatr Diabetes. 2019;20(5):530-7.

23. Dong B, Wang Z, Arnold LW, Song Y, Wang HJ, Ma J. Simplifying the screening of abdominal adiposity in Chinese children with waist-to-height ratio. Am J Hum Biol. 2016;28(6):945-9.

24. Fujita Y, Kouda K, Nakamura H, Iki M. Cut-off values of body mass index, waist circumference, and waist-to-height ratio to identify excess abdominal fat: population-based screening of Japanese school children. J Epidemiol. 2011;21(3):191-6.

25. Zhou D, Yang M, Yuan ZP, Zhang DD, Liang L, Wang CL, et al. Waist-to-Height Ratio: a simple, effective and practical screening tool for childhood obesity and metabolic syndrome. Prev Med. 2014;67:35-40.

26. Dai Y, Fu J, Liang L, Gong C, Xiong F, Liu G, et al. [A proposal for the cutoff point of waist-to-height for the diagnosis of metabolic syndrome in children and adolescents in six areas of China]. Zhonghua Liu Xing Bing Xue Za Zhi. 2014;35(8):882-5.

27. Matsha TE, Kengne AP, Yako YY, Hon GM, Hassan MS, Erasmus RT. Optimal waist-to-height ratio values for cardiometabolic risk screening in an ethnically diverse sample of South African urban and rural school boys and girls. PLoS One. 2013;8(8):e71133.

28. Bauer KW, Marcus MD, El ghormli L, Ogden CL, Foster GD. Cardio-metabolic risk screening among adolescents: understanding the utility of body mass index, waist circumference and waist to height ratio. Pediatr Obes. 2015;10(5):329-37.

29. Liu XL, Yin FZ, Ma CP, Gao GQ, Ma CM, Wang R, et al. Waist-to-height ratio as a screening measure for identifying adolescents with hypertriglyceridemic waist phenotype. J Pediatr Endocrinol Metab. 2015;28(9-10):1079-83.

30. Seo JY, Kim JH. Validation of surrogate markers for metabolic syndrome and cardiometabolic risk factor clustering in children and adolescents: A nationwide population-based study. PLoS One. 2017;12(10):e0186050.

31. Aguirre PF, Coca A, Aguirre MF, Celis G. Waist-to-height ratio and sedentary lifestyle as predictors of metabolic syndrome in children in Ecuador. Hipertens Riesgo Vasc. 2017.

32. Adegboye AR, Andersen LB, Froberg K, Sardinha LB, Heitmann BL. Linking definition of childhood and adolescent obesity to current health outcomes. Int J Pediatr Obes. 2010;5(2):130-42.

33. Ma CM, Yin FZ, Liu XL, Wang R, Lou DH, Lu Q. How to Simplify the Diagnostic Criteria of Metabolic Syndrome in Adolescents. Pediatr Neonatol. 2017;58(2):178-84.

34. Zhao M, Bovet P, Ma C, Xi B. Performance of different adiposity measures for predicting cardiovascular risk in adolescents. Sci Rep. 2017;7:43686.

35. Xu T, Liu J, Liu J, Zhu G, Han S. Relation between metabolic syndrome and body compositions among Chinese adolescents and adults from a large-scale population survey. BMC Public Health. 2017;17(1):337.

36. Oliveira RG, Guedes DP. Performance of anthropometric indicators as predictors of metabolic syndrome in Brazilian adolescents. BMC Pediatr. 2018;18(1):33.

37. Liu BY, Jiang Rh, Li P, Liu C, Li L. Cutoff Waist-to-height and Waist-to-hip Ratios for Metabolic Syndrome in Chinese Children and Adolescents. Journal of China Medical University. 2017;46(5):434-8,43.

38. Arsang-Jang S, Kelishadi R, Esmail Motlagh M, Heshmat R, Mansourian M. Temporal Trend of Non-Invasive Method Capacity for Early Detection of Metabolic Syndrome in Children and Adolescents: A Bayesian Multilevel Analysis of Pseudo-Panel Data. Ann Nutr Metab. 2019;75(1):55-65.

39. Vasquez F, Correa-Burrows P, Blanco E, Gahagan S, Burrows R. A waist-to-height ratio of 0.54 is a good predictor of metabolic syndrome in 16-year-old male and female adolescents. Pediatr Res. 2019;85(3):269-74.

40. Graves L, Garnett SP, Cowell CT, Baur LA, Ness A, Sattar N, et al. Waist-to-height ratio and cardiometabolic risk factors in adolescence: findings from a prospective birth cohort. Pediatr Obes. 2014;9(5):327-38.

41. Tompuri TT, Jaaskelainen J, Lindi V, Laaksonen DE, Eloranta AM, Viitasalo A, et al. Adiposity Criteria in Assessing Increased Cardiometabolic Risk in Prepubertal Children. Front Endocrinol (Lausanne). 2019;10:410.

42. Benmohammed K, Valensi P, Benlatreche M, Nguyen MT, Benmohammed F, Paries J, et al. Anthropometric markers for detection of the metabolic syndrome in adolescents. Diabetes Metab. 2015;41(2):138-44.

43. Yuan Y, Xie H, Sun L, Wang B, Zhang L, Han H, et al. A Novel Indicator of Children's Lipid Accumulation Product Associated with Impaired Fasting Glucose in Chinese Children and Adolescents. Diabetes Metab Syndr Obes. 2020;13:1653-60.

44. Zhang Y, Hu J, Li Z, Li T, Chen M, Wu L, et al. A Novel Indicator Of Lipid Accumulation Product Associated With Metabolic Syndrome In Chinese Children And Adolescents. Diabetes Metab Syndr Obes. 2019;12:2075-83.

45. Wang Y, Liu W, Sun L, Zhang Y, Wang B, Yuan Y, et al. A novel indicator, childhood lipid accumulation product, is associated with hypertension in Chinese children and adolescents. Hypertens Res. 2020;43(4):305-12.

46. Tee JYH, Gan WY, Lim PY. Comparisons of body mass index, waist circumference, waist-to-height ratio and a body shape index (ABSI) in predicting high blood pressure among Malaysian adolescents: a cross-sectional study. BMJ Open. 2020;10(1):e032874.

47. Vaquero-Álvarez M, Molina-Luque R, Fonseca-Pozo FJ, Molina-Recio G, López-Miranda J, Romero-Saldaña M. Diagnostic Precision of Anthropometric Variables for the Detection of Hypertension in Children and Adolescents. Int J Environ Res Public Health. 2020;17(12).

48. Cristine Silva K, Santana Paiva N, Rocha de Faria F, Franceschini S, Eloiza Piore S. Predictive Ability of Seven Anthropometric Indices for Cardiovascular Risk Markers and Metabolic Syndrome in Adolescents. J Adolesc Health. 2020;66(4):491-8.

49. Arellano-Ruiz P, García-Hermoso A, García-Prieto JC, Sánchez-López M, Vizcaíno VM, Solera-Martínez M. Predictive Ability of Waist Circumference and Waist-to-Height Ratio for Cardiometabolic Risk Screening among Spanish Children. Nutrients. 2020;12(2).

50. Li Y, Zou Z, Luo J, Ma J, Ma Y, Jing J, et al. The predictive value of anthropometric indices for cardiometabolic risk factors in Chinese children and adolescents: A national multicenter school-based study. PLoS One. 2020;15(1):e0227954.

51. Mai TMT, Gallegos D, Jones L, Tran QC, Tran TMH, van der Pols JC. The utility of anthopometric indicators to identify cardiovascular risk factors in Vietnamese children. Br J Nutr. 2020;123(9):1043-55.

52. Yazdi M, Assadi F, Qorbani M, Daniali SS, Heshmat R, Esmaeil Motlagh M, et al. Validity of anthropometric indices in predicting high blood pressure risk factors in Iranian children and adolescents: CASPIAN-V study. J Clin Hypertens (Greenwich). 2020;22(6):1009-17.

53. Kilinc A, Col N, Demircioglu-Kilic B, Aydin N, Balat A, Keskin M. Waist to height ratio as a screening tool for identifying childhood obesity and associated factors. Pak J Med Sci. 2019;35(6):1652-8.
