# Supplementary material for: Performance of waist-to-height ratio as a screening tool for identifying cardiometabolic risk in children: a meta-analysis
Source: Diabetol Metab Syndr. 2021 Jun 14;13:66. doi: 10.1186/s13098-021-00688-7 (PMC8201900; doi:10.1186/s13098-021-00688-7)
Supplement: Supplementary file 4 — Additional file 4: Table S3. Other pooled results of WHtR screening for CMRs in children and adolescents. [file 13098_2021_688_MOESM4_ESM.docx]

**Table S3. Other pooled results of WHtR screening for CMRs in children and adolescents**

| Outcomes | Population | *I^2^* | *Q* statistic | *P*-value | PLR (95% *CI*) | NLR (95% *CI*) | Correlation Coefficient (95% *CI*) of Deek’s Funnel Plot Asymmetry Test | *t* | *P*-value | |  |
| --- | --- | --- | --- | --- | --- | --- | --- | --- | --- | --- | --- |
| CMR_3_ | 99331 | 100 (100-100) | 1012.78 | ＜0.001 | 5.20 (3.70, 7.30) | 0.19(0.12, 0.30) | -7.20 (-50.60, 36.21) | -0.34 | | 0.734 | |
| CMR_2_ | 46448 | 100 (100-100) | 1190.41 | ＜0.001 | 3.50 (2.40, 5.20) | 0.22(0.10, 0.51) | 19.38 (-49.64, 88.40) | 0.66 | | 0.528 | |
| CMR_1_ | 20268 | 100 (99-100) | 522.89 | ＜0.001 | 3.50 (1.90, 6.40) | 0.54(0.40, 0.71) | -73.53 (-546.28, 399.23) | -0.67 | | 0.572 | |
| Elevated FBG | 13749 | 100 (100-100) | 1012.05 | ＜0.001 | 1.40 (1.00, 2.00) | 0.75(0.62, 0.90) | 10.43 (-9.86, 30.72) | 1.26 | | 0.255 | |
| Elevated BP | 101786 | 100 (100-100) | 6020.87 | ＜0.001 | 2.10 (1.80, 2.50) | 0.61(0.54, 0.70) | 15.78 (1.46, 30.10) | 2.30 | | 0.032 | |
| Dyslipidaemia | 73092 | 100 (100-100) | 1704.56 | ＜0.001 | 1.70 (1.50,2.00) | 0.67(0.56, 0.79) | 6.64 (-3.89, 17.16) | 1.32 | | 0.202 | |
| Elevated TG | 12599 | 100 (99-100) | 476.77 | ＜0.001 | 2.00 (1.50, 2.60) | 0.60(0.39, 0.91) | -1.62 (-49.58, 46.34) | -0.09 | | 0.930 | |
| Low HDL-C | 12604 | 100 (99-100) | 410.18 | ＜0.001 | 1.90 (1.60, 2.40) | 0.67(0.44, 1.03) | 10.90 (-34.55, 56.34) | 0.67 | | 0.542 | |
| Central obesity | 148115 | 100 (100-100) | 964.57 | ＜0.001 | 8.80 (6.20, 12.70) | 0.10(0.06, 0.17) | 58.57 (-42.61, 159.76) | 1.49 | | 0.197 | |

WHtR, waist-to-height ratio; CMR: cardiometabolic risk factor; CMR_3:_ presenting with at least three CMRs; CMR_2_: presenting with at least two CMRs; CMR_1_: presenting with at least one of CMRs; FBG, fasting blood glucose; BP, blood pressure; TG, triglyceride; HDL-C, High-density leptin cholesterol; PLR, positive likelihood ratio; NLR, negative likelihood ratio; CI: confidence interval.

The results of pooled elevated total cholesterol, high low-density leptin cholesterol, and SBP/DBP blood pressure were not reported.
