# Supplementary figures and images for: Performance of waist-to-height ratio as a screening tool for identifying cardiometabolic risk in children: a meta-analysis
Source: Diabetol Metab Syndr. 2021 Jun 14;13:66. doi: 10.1186/s13098-021-00688-7 (PMC8201900; doi:10.1186/s13098-021-00688-7)

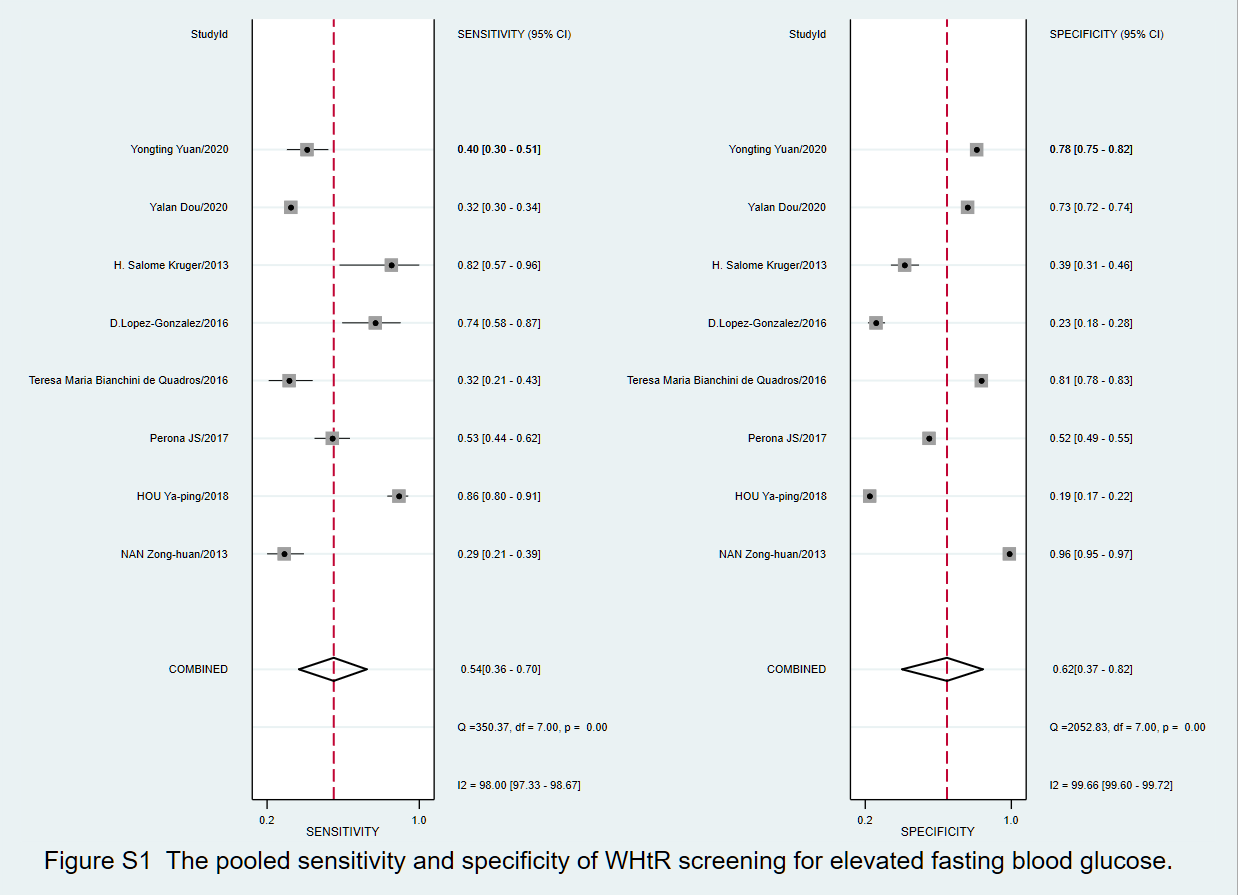


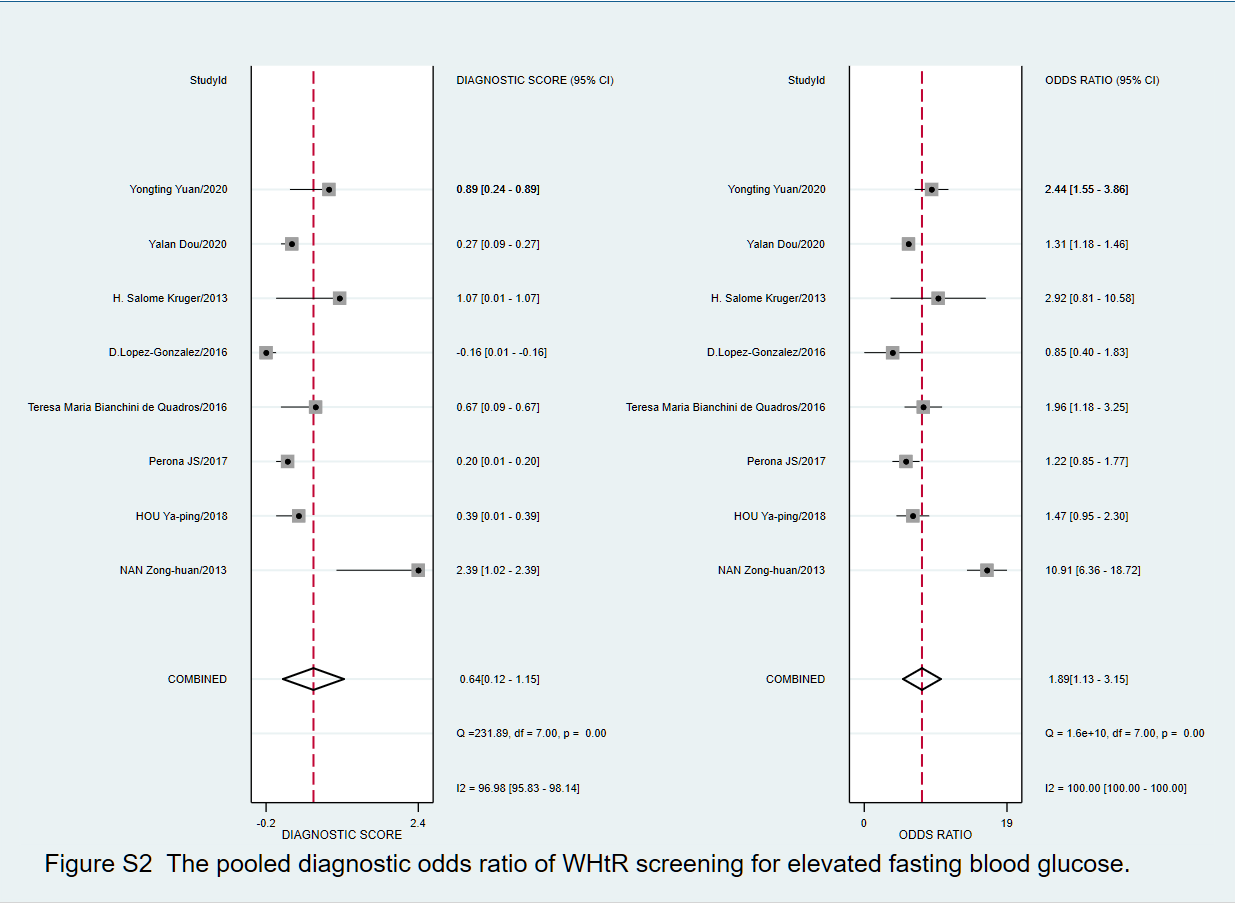


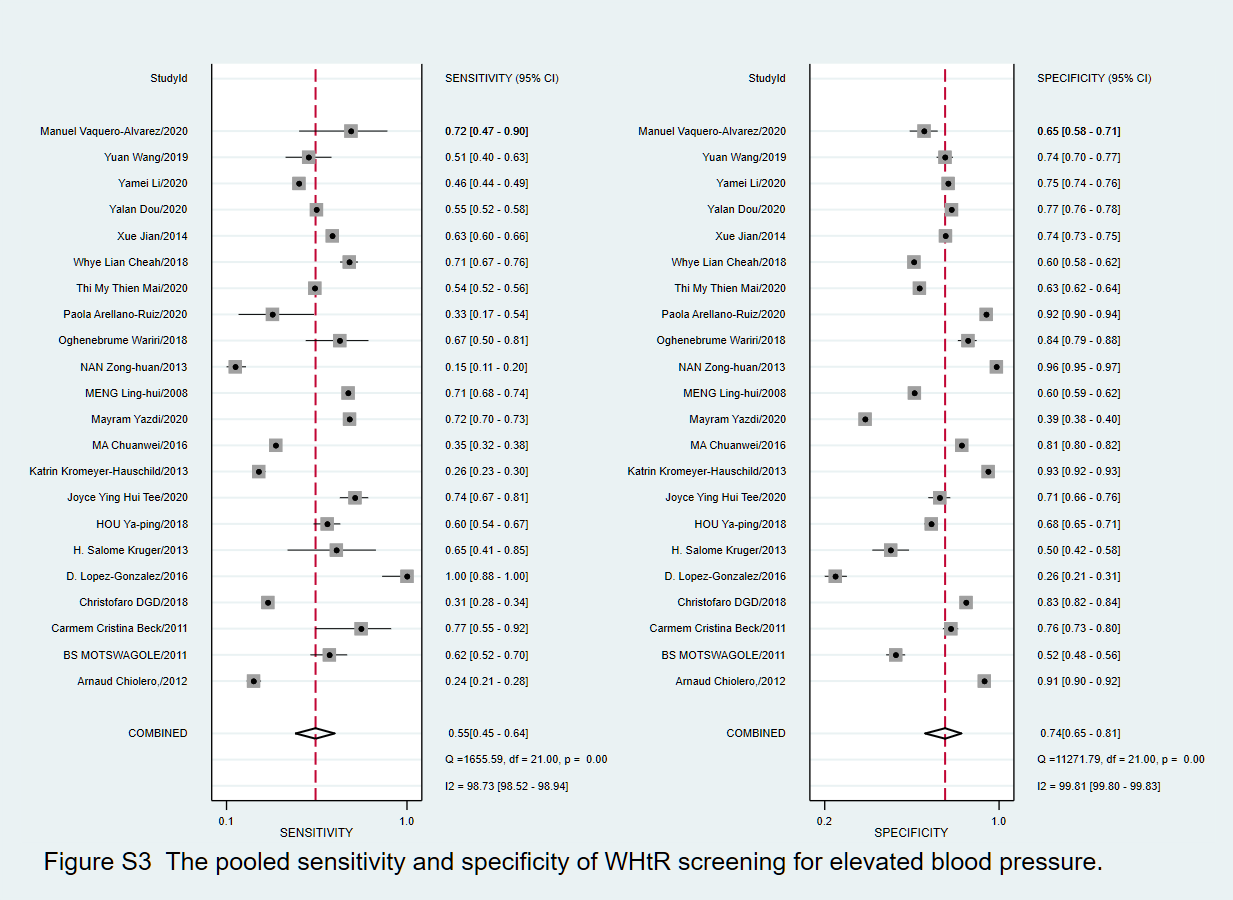


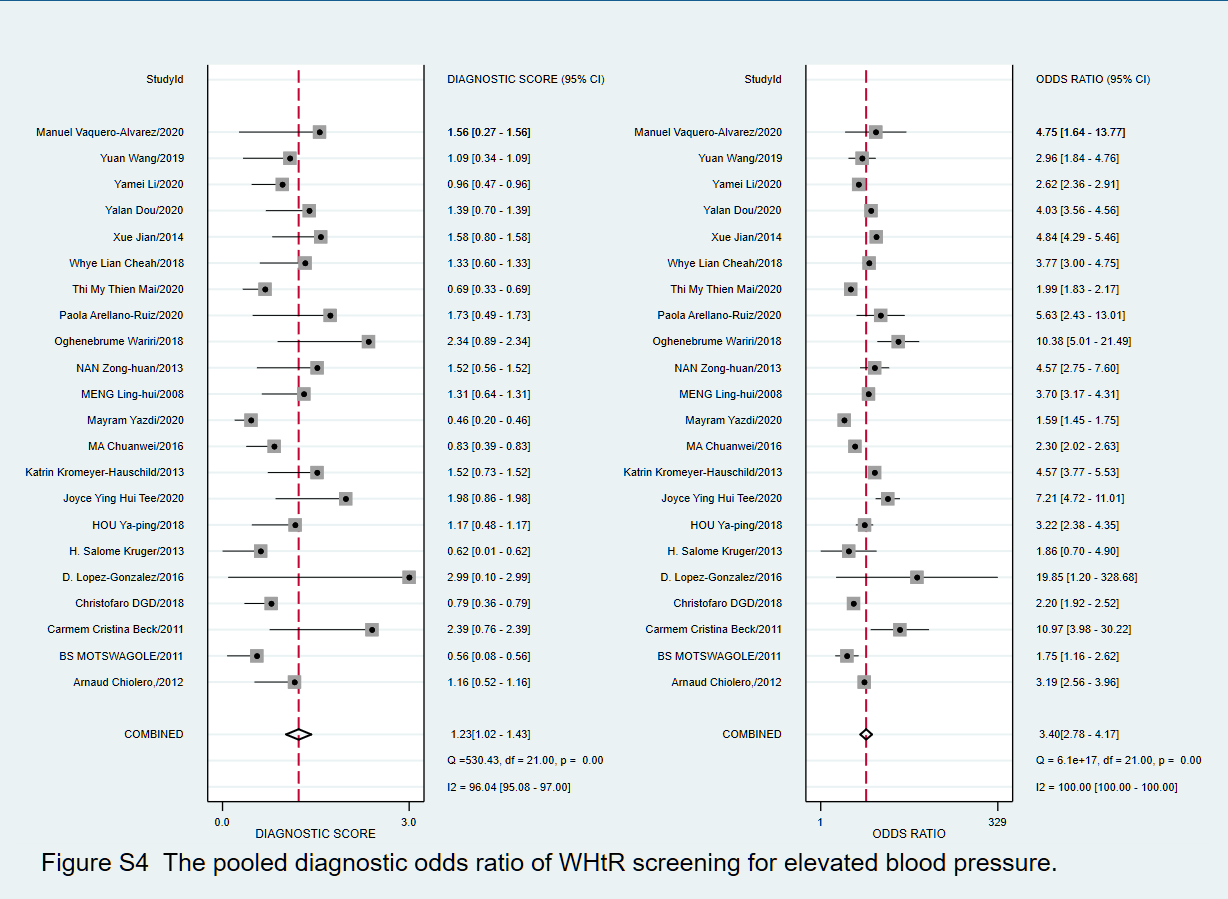


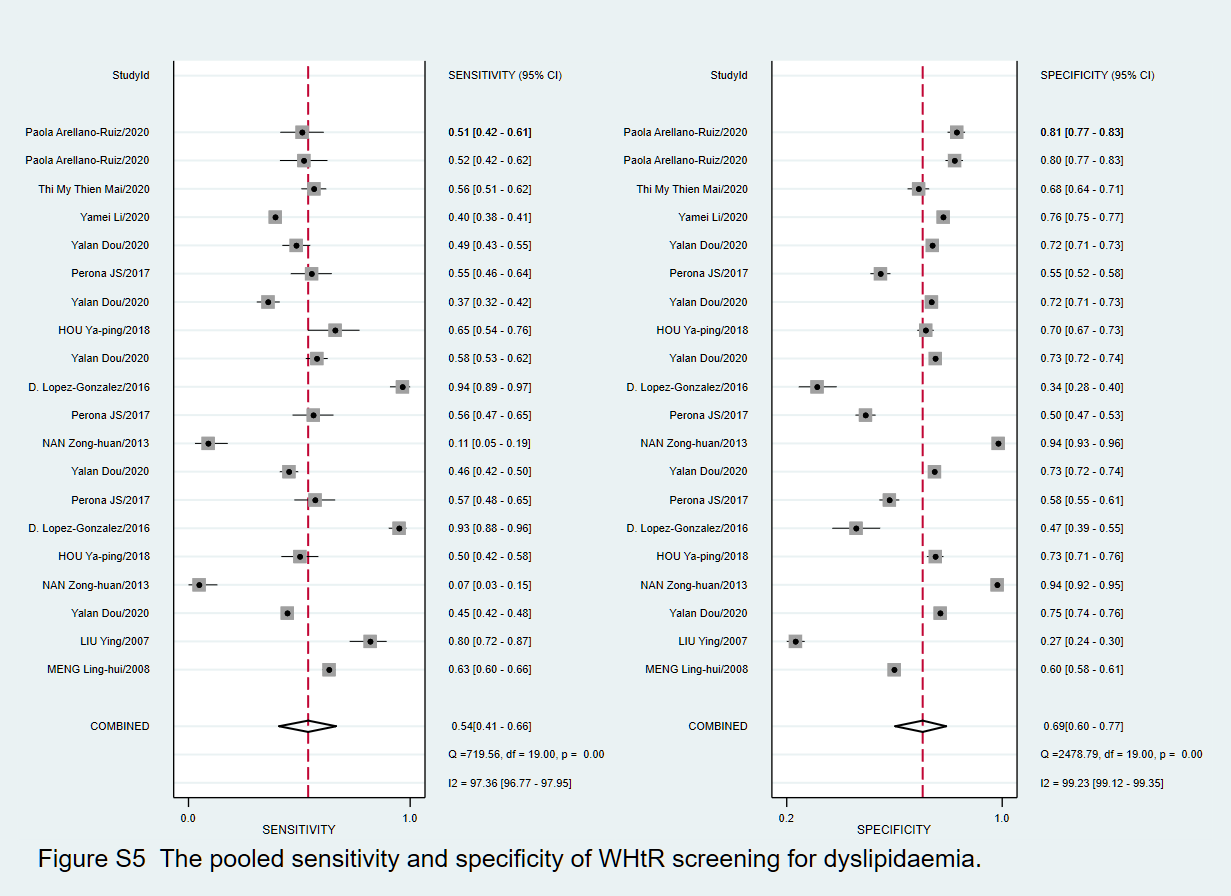


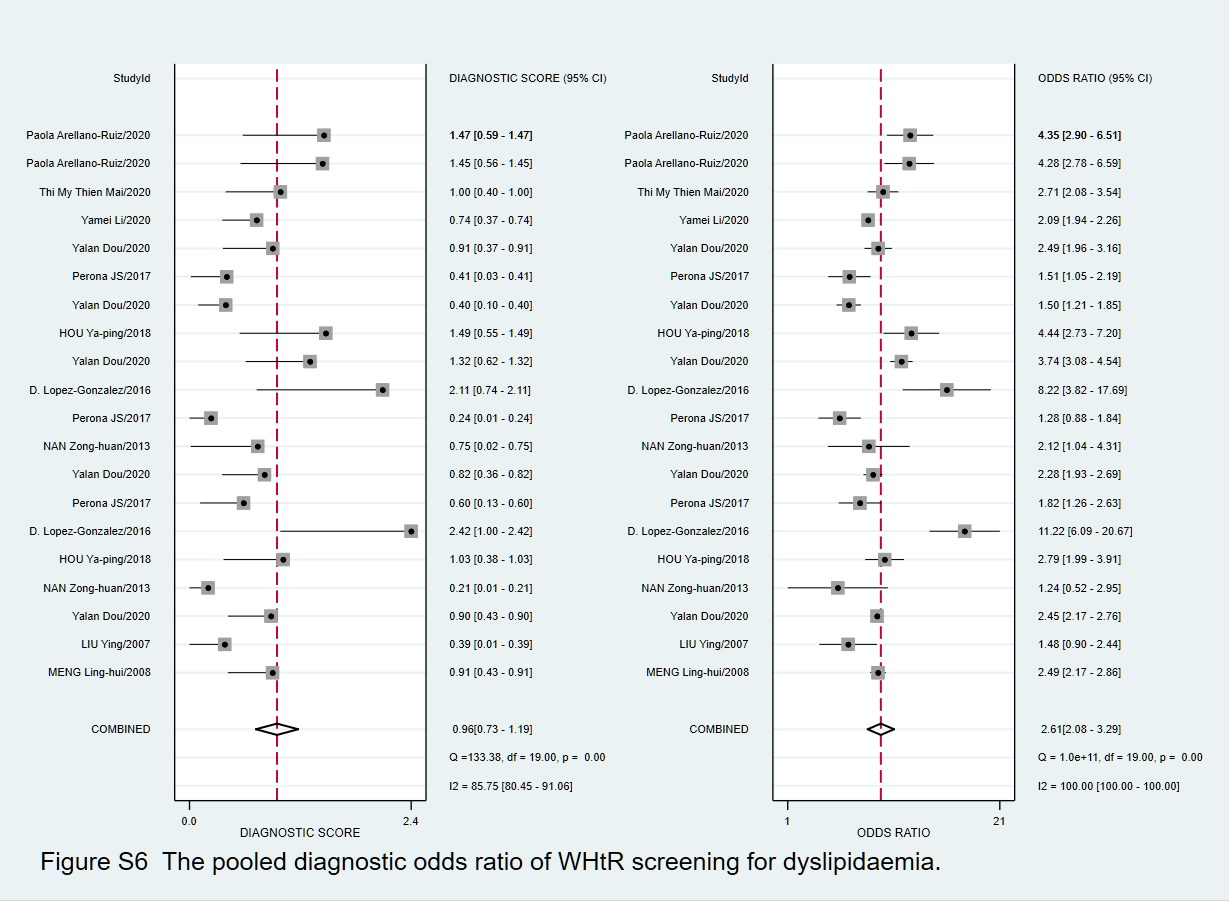


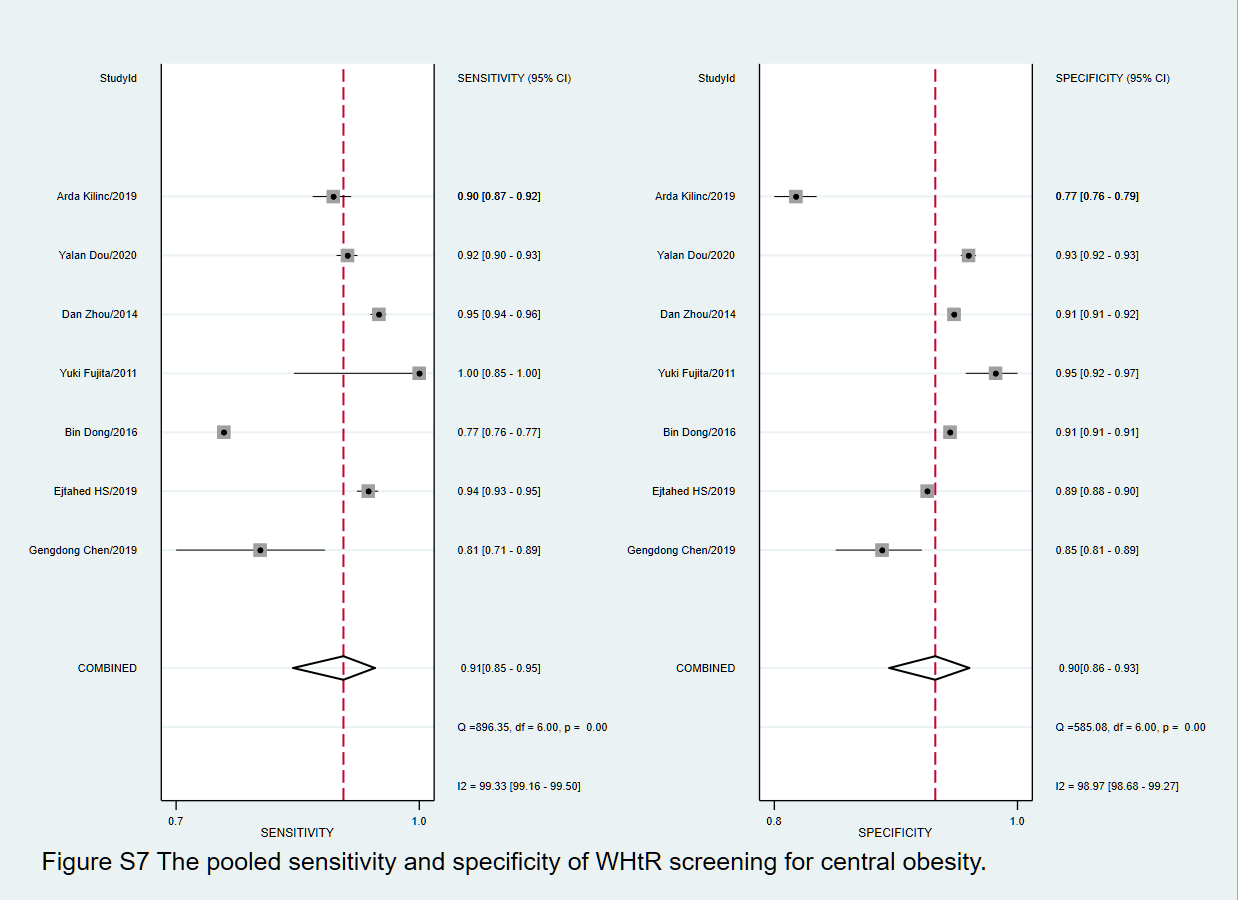


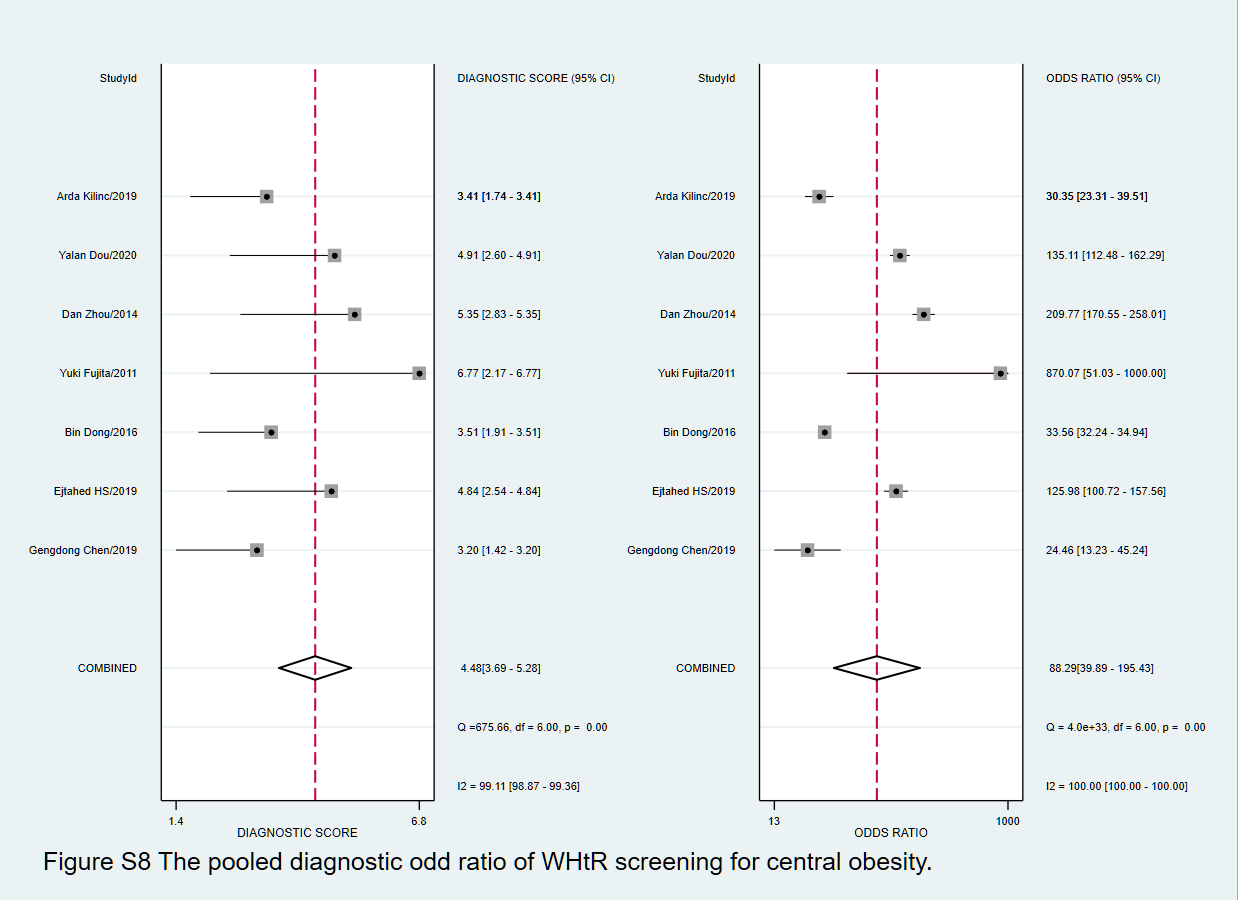


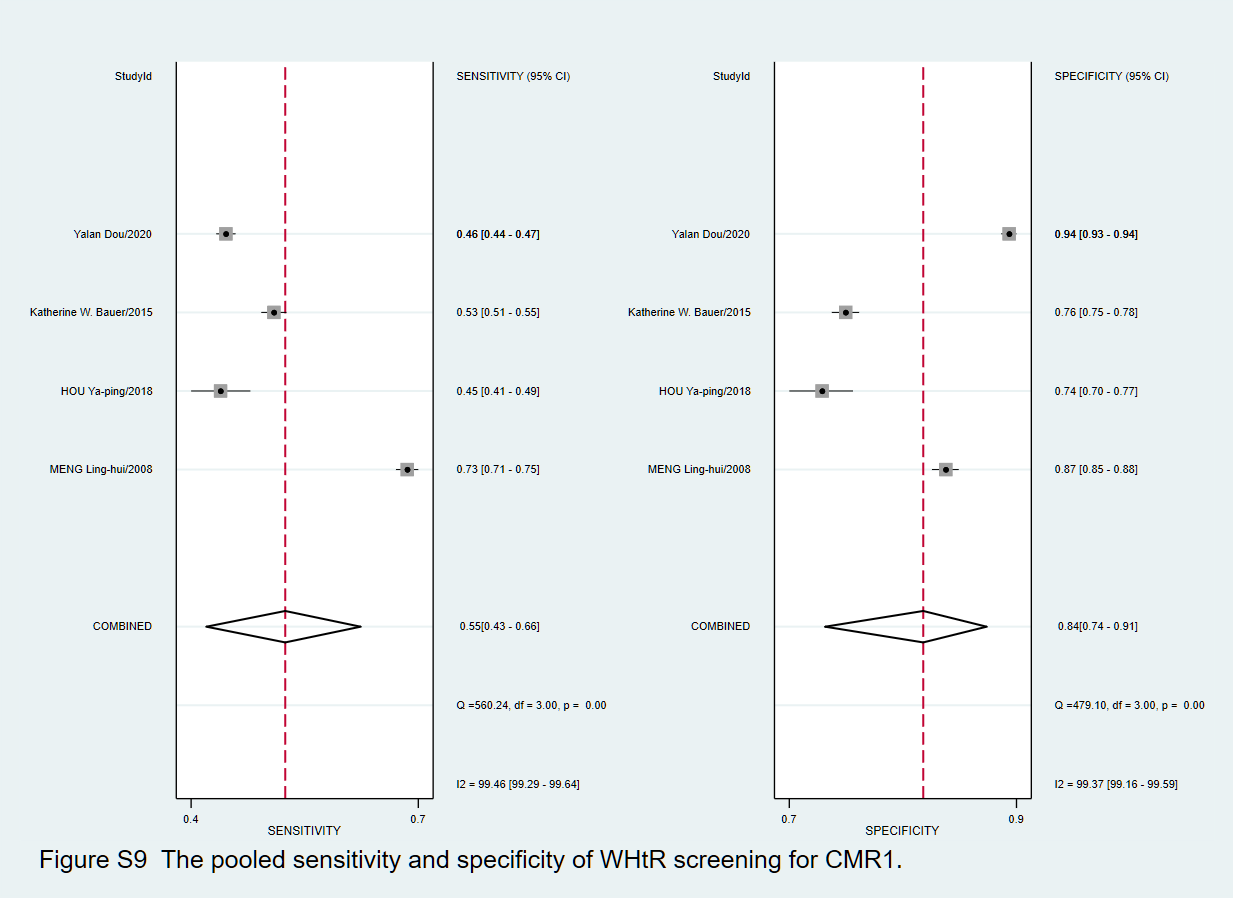


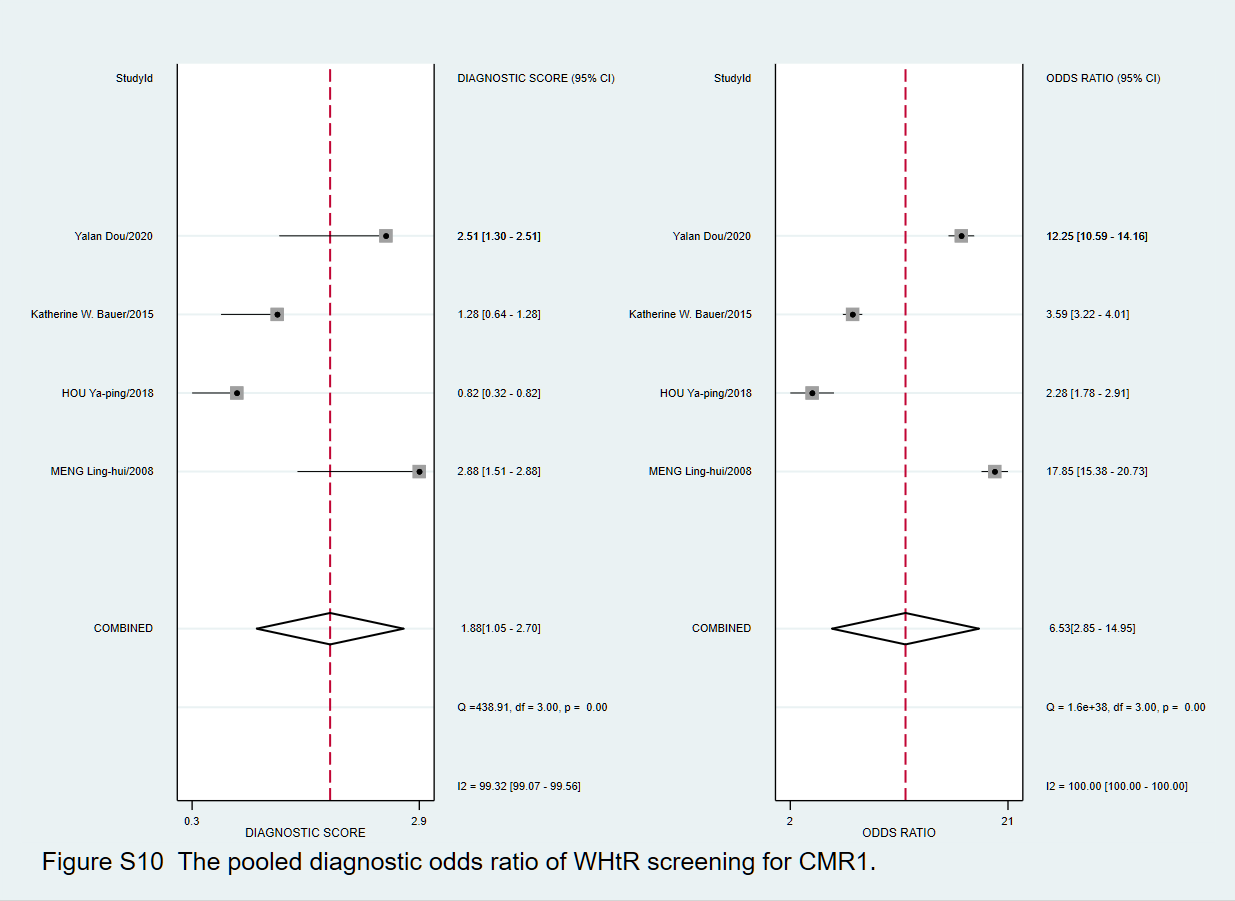


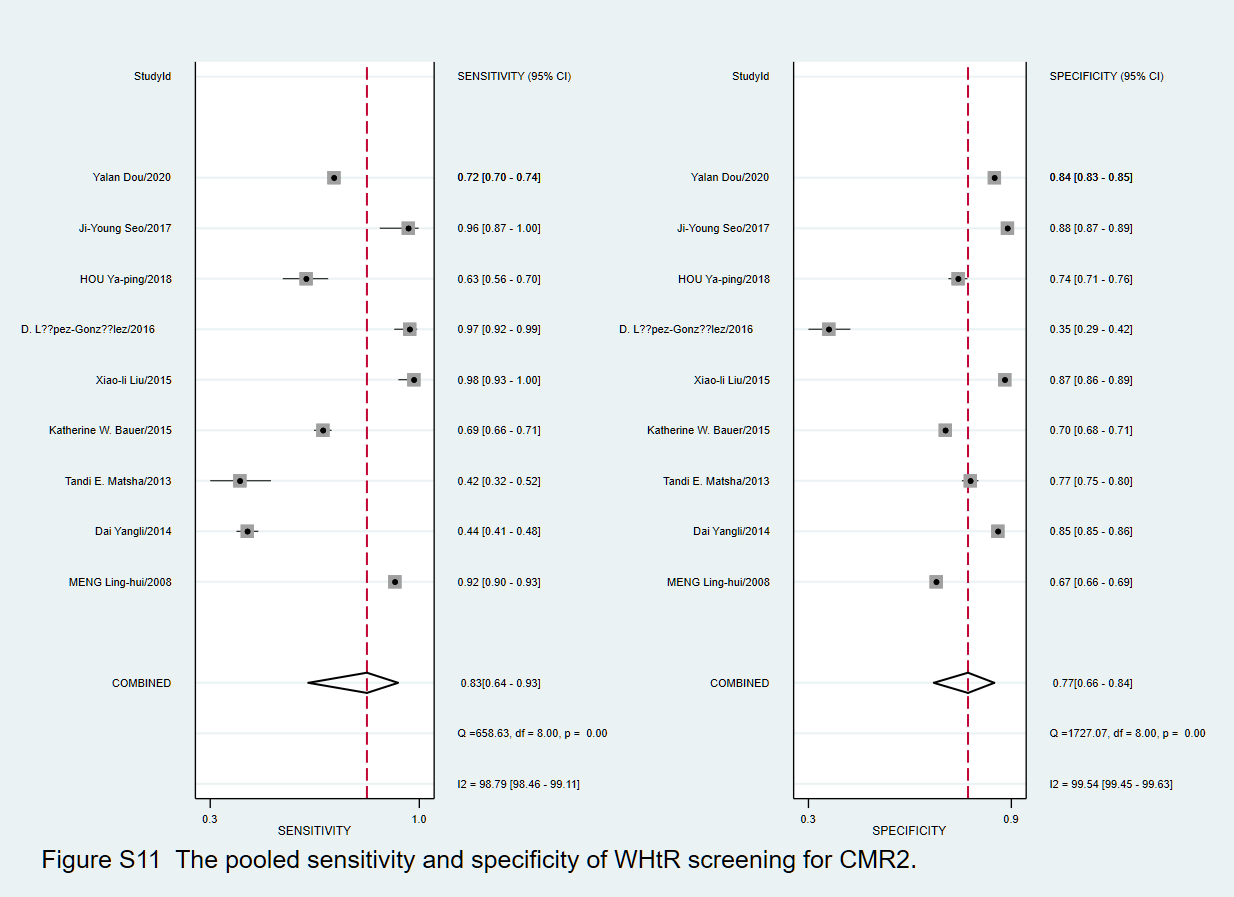


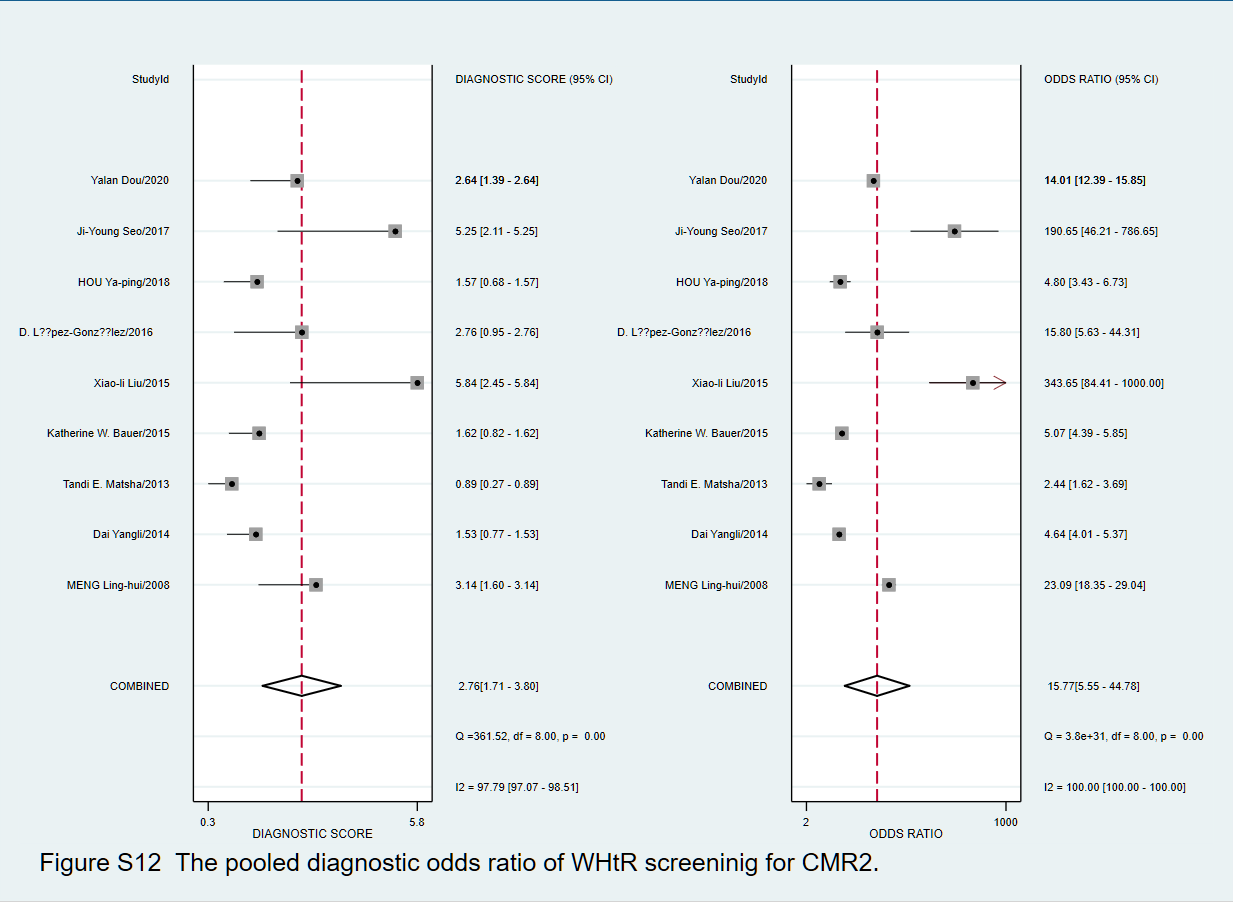


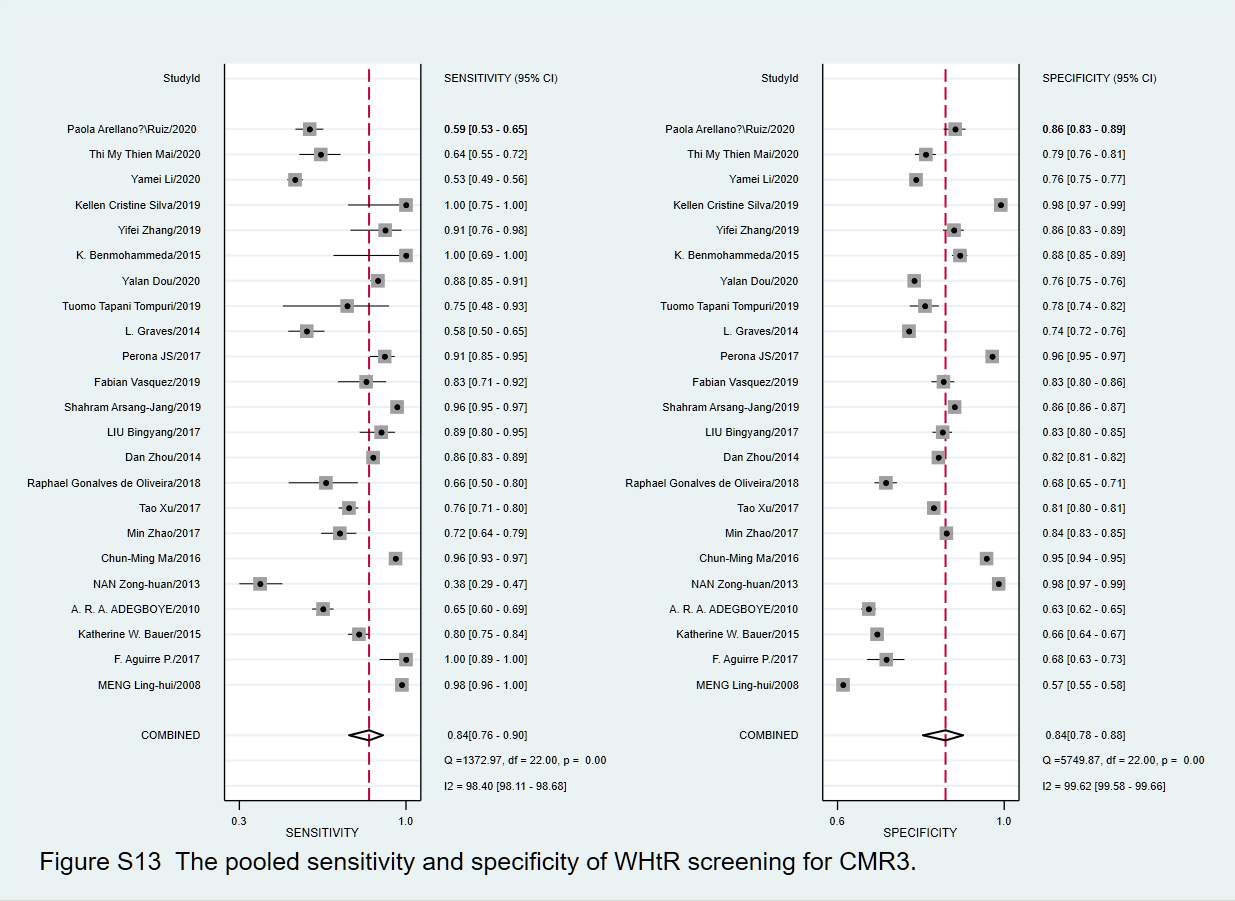


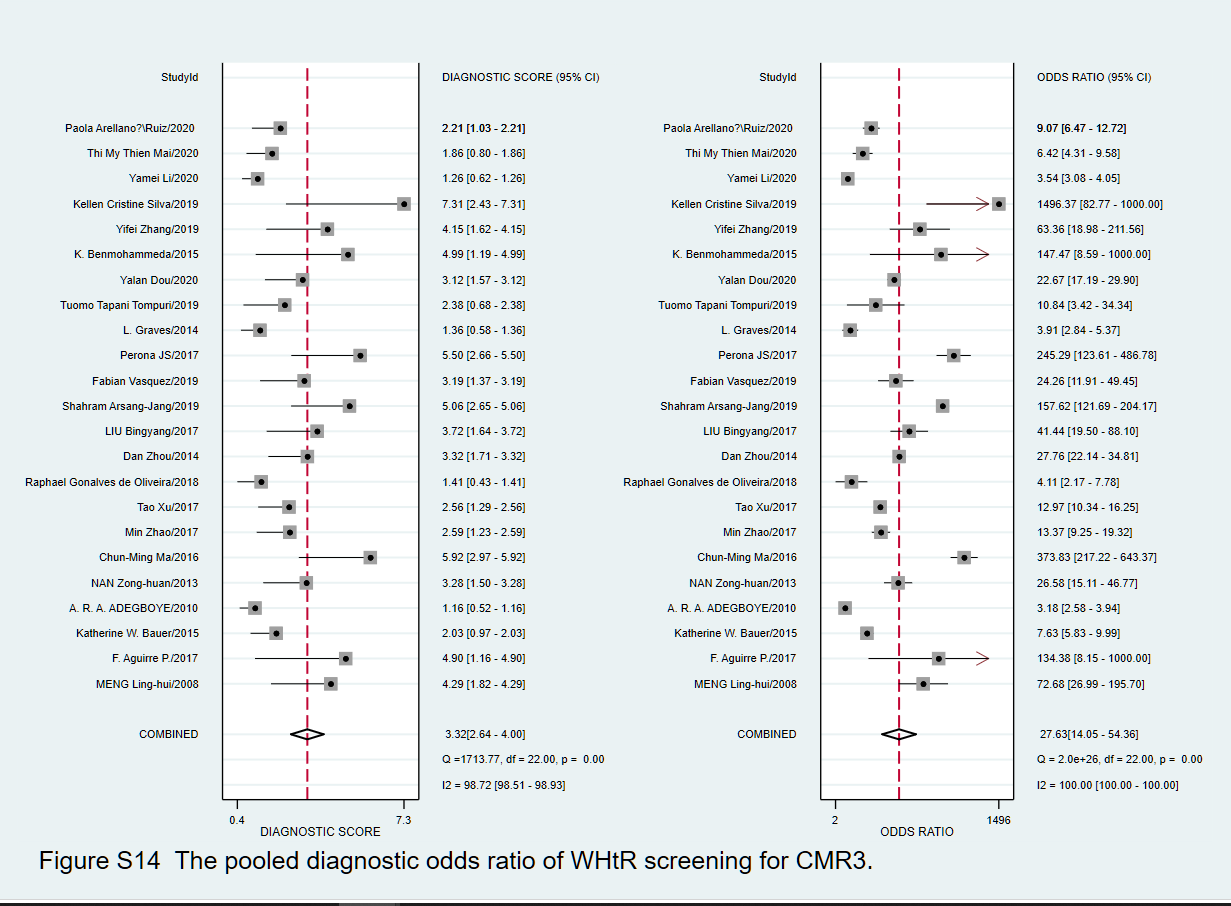

Supplement: Supplementary file 5 — Additional file 5: Figure S1-S14. The forest plots of pooled sensitivities, specificities, and odd diagnostic ratios in discriminating CMRs. [file 13098_2021_688_MOESM5_ESM.docx]
