# Supplementary material for: Performance of waist-to-height ratio as a screening tool for identifying cardiometabolic risk in children: a meta-analysis
Source: Diabetol Metab Syndr. 2021 Jun 14;13:66. doi: 10.1186/s13098-021-00688-7 (PMC8201900; doi:10.1186/s13098-021-00688-7)
Supplement: Supplementary file 6 — Additional file 6: Table S4. Pooled results of WHtR screening for CMRs by sex in children and adolescents. [file 13098_2021_688_MOESM6_ESM.docx]

**Table S4. Pooled results of WHtR screening for CMRs by sex in children and adolescents**

| Outcome | Sex | Population | Prevalence | *Q* statistic | *P*-value | *I^2^* | Threshold effect  (correlation coefficient) | *P*-value |
| --- | --- | --- | --- | --- | --- | --- | --- | --- |
| CMR_3_ (1-18) | Boy | 44271 | 0.07 | 363.86 | ＜0.001 | 99 (99-100) | -0.55 | 0.019 |
|  | Girl | 41546 | 0.06 | 141.96 | ＜0.001 | 99 (98-99) | -0.63 | 0.005 |
| CMR_2_ (1, 19-21) | Boy | 16193 | 0.11 | 116.44 | ＜0.001 | 98 (97-99) | -0.12 | 0.885 |
|  | Girl | 14874 | 0.07 | 80.53 | ＜0.001 | 98 (96-99) | -0.47 | 0.534 |
| Elevated BP (1-3, 22-29) | Boy | 39924 | 0.17 | 2356.13 | ＜0.001 | 100 (100-100) | 0.67 | 0.026 |
|  | Girl | 38535 | 0.14 | 2483.41 | ＜0.001 | 100 (100-100) | 0.43 | 0.188 |
| Dyslipidaemia (1-3, 8, 30) | Boy | 31900 | 0.13 | 388.41 | ＜0.001 | 99 (99-100) | 0.61 | 0.048 |
|  | Girl | 28832 | 0.14 | 135.36 | ＜0.001 | 99 (98-99) | 0.73 | 0.016 |
| Central obesity (1, 12, 31-35) | Boy | 82571 | 0.26 | 50.14 | ＜0.001 | 96 (93-99) | -0.24 | 0.600 |
|  | Girl | 81324 | 0.26 | 516.56 | ＜0.001 | 100 (99-100) | -0.42 | 0.351 |

WHtR, waist-to-height ratio; CMR: cardiometabolic risk factors; CMR_3_: presenting with three or more CMRs; CMR_2_: presenting with two or more CMRs; BP, blood pressure; AUSROC, area under the summary receiver operating characteristic; PLR, positive likelihood ratio; NLR, negative likelihood ratio; DOR, diagnostic odds ratio; CI: confidence interval.

The results of pooled elevated fasting blood glucose, total cholesterol, triglyceride, low high-density leptin cholesterol, elevated low-density leptin cholesterol, SBP/DBP blood pressure and CMR_1_ were not shown.

(Continued)

| Outcomes | Sex | AUSROC (95% *CI*) |  | Sensitivity (95% *CI*) | Specificity (95% *CI*) | PLR (95% *CI*) |
| --- | --- | --- | --- | --- | --- | --- |
| CMR_3_ | Boy | 0.91 (0.89, 0.93) |  | 0.89 (0.78, 0.95) | 0.83 (0.77, 0.88) | 5.30 (3.60, 7.80) |
|  | Girl | 0.90 (0.88, 0.93) |  | 0.84 (0.76, 0.90) | 0.83 (0.76, 0.88) | 5.00 (3.30, 7.60) |
| CMR_2_ | Boy | 0.87 (0.84, 0.89) |  | 0.75 (0.36, 0.94) | 0.86 (0.85, 0.87) | 5.50 (3.40, 8.80) |
|  | Girl | 0.84 (0.81, 0.87) |  | 0.71 (0.36, 0.91) | 0.82 (0.75, 0.87) | 4.00 (2.10, 7.60 ) |
| Elevated BP | Boy | 0.70 (0.65, 0.73) |  | 0.61 (0.52, 0.69) | 0.70 (0.59, 0.79) | 2.00 (1.60, 2.60) |
|  | Girl | 0.66 (0.62, 0.70) |  | 0.52 (0.41, 0.63) | 0.73 (0.62, 0.82) | 1.90 (1.50, 2.40) |
| Dyslipidaemia | Boy | 0.65 (0.61, 0.69) |  | 0.57 (0.50, 0.63) | 0.67 (0.60, 0.73) | 1.70 (1.50, 1.90) |
|  | Girl | 0.58 (0.54, 0.62) |  | 0.44 (0.38, 0.50) | 0.70 (0.64, 0.75) | 1.50 (1.30, 1.70) |
| Central obesity | Boy | 0.96 (0.94, 0.97) |  | 0.92 (0.90, 0.93) | 0.90 (0.87, 0.93) | 9.50 (6.90, 13.10) |
|  | Girl | 0.96 (0.94, 0.98) |  | 0.92 (0.87, 0.95) | 0.89 (0.84, 0.93) | 8.40 (5.60, 12.70) |

(Continued)

| Outcomes | Sex | NLR (95% *CI*) | DOR (95% *CI*) | Correlation Coefficient (95% *CI*) of Deek’s Funnel Plot Asymmetry Test | *t* | *P*-value |
| --- | --- | --- | --- | --- | --- | --- |
| CMR_3_ | Boy | 0.14 (0.07, 0.29) | 39 (14, 110) | -33.55 (-101.07, 33.97) | -1.05 | 0.308 |
|  | Girl | 0.19 (0.11, 0.31) | 26 (11, 64) | -4.92 (-39.25, 29.41) | -0.3 | 0.765 |
| CMR_2_ | Boy | 0.29 (0.08, 1.02) | 19 (3, 105) | 21.46 (-172.54, 215.46) | 0.48 | 0.681 |
|  | Girl | 0.35 (0.12, 1.03) | 11 (2, 62) | 13.86 (-163.04, 190.75) | 0.34 | 0.768 |
| Elevated BP | Boy | 0.56 (0.48, 0.65) | 4 (3, 5) | 12.35 (-22.11, 46.79) | 0.81 | 0.439 |
|  | Girl | 0.66 (0.57, 0.76) | 3 (2, 4) | 20.45 (0.74, 40.17) | 2.35 | 0.043 |
| Dyslipidaemia | Boy | 0.65 (0.58, 0.73) | 3 (2, 3) | -0.82 (-10.65, 9.00) | -0.19 | 0.854 |
|  | Girl | 0.80 (0.74, 0.86) | 2 (1, 2) | 2.01 (-11.04, 15.05) | 0.35 | 0.732 |
| Central obesity | Boy | 0.09 (0.07, 0.11) | 106 (71, 158) | 12.37 (-24.93, 49.67) | 0.85 | 0.433 |
|  | Girl | 0.09 (0.05, 0.14) | 96 (45, 206) | 20.16 (-31.89, 72.22) | 1 | 0.365 |

**References**

1. Dou Y, Jiang Y, Yan Y, Chen H, Zhang Y, Chen X, et al. Waist-to-height ratio as a screening tool for cardiometabolic risk in children and adolescents: a nationwide cross-sectional study in China. BMJ Open. 2020;10(6):e037040.

2. Li Y, Zou Z, Luo J, Ma J, Ma Y, Jing J, et al. The predictive value of anthropometric indices for cardiometabolic risk factors in Chinese children and adolescents: A national multicenter school-based study. PLoS One. 2020;15(1):e0227954.

3. Mai TMT, Gallegos D, Jones L, Tran QC, Tran TMH, van der Pols JC. The utility of anthopometric indicators to identify cardiovascular risk factors in Vietnamese children. Br J Nutr. 2020;123(9):1043-55.

4. Cristine Silva K, Santana Paiva N, Rocha de Faria F, Franceschini S, Eloiza Piore S. Predictive Ability of Seven Anthropometric Indices for Cardiovascular Risk Markers and Metabolic Syndrome in Adolescents. J Adolesc Health. 2020;66(4):491-8.

5. Benmohammed K, Valensi P, Benlatreche M, Nguyen MT, Benmohammed F, Paries J, et al. Anthropometric markers for detection of the metabolic syndrome in adolescents. Diabetes Metab. 2015;41(2):138-44.

6. Tompuri TT, Jaaskelainen J, Lindi V, Laaksonen DE, Eloranta AM, Viitasalo A, et al. Adiposity Criteria in Assessing Increased Cardiometabolic Risk in Prepubertal Children. Front Endocrinol (Lausanne). 2019;10:410.

7. Graves L, Garnett SP, Cowell CT, Baur LA, Ness A, Sattar N, et al. Waist-to-height ratio and cardiometabolic risk factors in adolescence: findings from a prospective birth cohort. Pediatr Obes. 2014;9(5):327-38.

8. Perona JS, Schmidt-RioValle J, Rueda-Medina B, Correa-Rodriguez M, Gonzalez-Jimenez E. Waist circumference shows the highest predictive value for metabolic syndrome, and waist-to-hip ratio for its components, in Spanish adolescents. Nutr Res. 2017;45:38-45.

9. Arsang-Jang S, Kelishadi R, Esmail Motlagh M, Heshmat R, Mansourian M. Temporal Trend of Non-Invasive Method Capacity for Early Detection of Metabolic Syndrome in Children and Adolescents: A Bayesian Multilevel Analysis of Pseudo-Panel Data. Ann Nutr Metab. 2019;75(1):55-65.

10. Vasquez F, Correa-Burrows P, Blanco E, Gahagan S, Burrows R. A waist-to-height ratio of 0.54 is a good predictor of metabolic syndrome in 16-year-old male and female adolescents. Pediatr Res. 2019;85(3):269-74.

11. Liu BY, Jiang Rh, Li P, Liu C, Li L. Cutoff Waist-to-height and Waist-to-hip Ratios for Metabolic Syndrome in Chinese Children and Adolescents. Journal of China Medical University. 2017;46(5):434-8,43.

12. Zhou D, Yang M, Yuan ZP, Zhang DD, Liang L, Wang CL, et al. Waist-to-Height Ratio: a simple, effective and practical screening tool for childhood obesity and metabolic syndrome. Prev Med. 2014;67:35-40.

13. Oliveira RG, Guedes DP. Performance of anthropometric indicators as predictors of metabolic syndrome in Brazilian adolescents. BMC Pediatr. 2018;18(1):33.

14. Xu T, Liu J, Liu J, Zhu G, Han S. Relation between metabolic syndrome and body compositions among Chinese adolescents and adults from a large-scale population survey. BMC Public Health. 2017;17(1):337.

15. Zhao M, Bovet P, Ma C, Xi B. Performance of different adiposity measures for predicting cardiovascular risk in adolescents. Sci Rep. 2017;7:43686.

16. Ma CM, Yin FZ, Liu XL, Wang R, Lou DH, Lu Q. How to Simplify the Diagnostic Criteria of Metabolic Syndrome in Adolescents. Pediatr Neonatol. 2017;58(2):178-84.

17. Adegboye AR, Andersen LB, Froberg K, Sardinha LB, Heitmann BL. Linking definition of childhood and adolescent obesity to current health outcomes. Int J Pediatr Obes. 2010;5(2):130-42.

18. Aguirre PF, Coca A, Aguirre MF, Celis G. Waist-to-height ratio and sedentary lifestyle as predictors of metabolic syndrome in children in Ecuador. Hipertens Riesgo Vasc. 2017.

19. Liu XL, Yin FZ, Ma CP, Gao GQ, Ma CM, Wang R, et al. Waist-to-height ratio as a screening measure for identifying adolescents with hypertriglyceridemic waist phenotype. J Pediatr Endocrinol Metab. 2015;28(9-10):1079-83.

20. Matsha TE, Kengne AP, Yako YY, Hon GM, Hassan MS, Erasmus RT. Optimal waist-to-height ratio values for cardiometabolic risk screening in an ethnically diverse sample of South African urban and rural school boys and girls. PLoS One. 2013;8(8):e71133.

21. Dai Y, Fu J, Liang L, Gong C, Xiong F, Liu G, et al. [A proposal for the cutoff point of waist-to-height for the diagnosis of metabolic syndrome in children and adolescents in six areas of China]. Zhonghua Liu Xing Bing Xue Za Zhi. 2014;35(8):882-5.

22. Cheah WL, Chang CT, Hazmi H, Kho GWF. Using Anthropometric Indicator to Identify Hypertension in Adolescents: A Study in Sarawak, Malaysia. Int J Hypertens. 2018;2018:6736251.

23. Xue J. The predictive effect of obesity-related indicators and blood pressure to height ratio on hypertension among urban school-age children: Shandong University; 2014.

24. Kromeyer-Hauschild K, Neuhauser H, Schaffrath Rosario A, Schienkiewitz A. Abdominal obesity in German adolescents defined by waist-to-height ratio and its association to elevated blood pressure: the KiGGS study. Obes Facts. 2013;6(2):165-75.

25. Motswagole BS, Kruger HS, Faber M, van Rooyen JM, de Ridder JH. The sensitivity of waist-to-height ratio in identifying children with high blood pressure. Cardiovasc J Afr. 2011;22(4):208-11.

26. Tee JYH, Gan WY, Lim PY. Comparisons of body mass index, waist circumference, waist-to-height ratio and a body shape index (ABSI) in predicting high blood pressure among Malaysian adolescents: a cross-sectional study. BMJ Open. 2020;10(1):e032874.

27. Yazdi M, Assadi F, Qorbani M, Daniali SS, Heshmat R, Esmaeil Motlagh M, et al. Validity of anthropometric indices in predicting high blood pressure risk factors in Iranian children and adolescents: CASPIAN-V study. J Clin Hypertens (Greenwich). 2020;22(6):1009-17.

28. Ma CW, Liang YJ, Xi B. Comparison of the performance of waist circumference and waist-height ratio in predicting elevated blood pressure among children and adolescents. Chinese Journal of School Health. 2016;37(10):1445-8.

29. Beck CC, Lopes Ada S, Pitanga FJ. Anthropometric indicators as predictors of high blood pressure in adolescents. Arq Bras Cardiol. 2011;96(2):126-33.

30. Zheng W, Zhao A, Xue Y, Zheng Y, Chen Y, Mu Z, et al. Gender and urban-rural difference in anthropometric indices predicting dyslipidemia in Chinese primary school children: a cross-sectional study. Lipids Health Dis. 2016;15:87.

31. Kilinc A, Col N, Demircioglu-Kilic B, Aydin N, Balat A, Keskin M. Waist to height ratio as a screening tool for identifying childhood obesity and associated factors. Pak J Med Sci. 2019;35(6):1652-8.

32. Fujita Y, Kouda K, Nakamura H, Iki M. Cut-off values of body mass index, waist circumference, and waist-to-height ratio to identify excess abdominal fat: population-based screening of Japanese school children. J Epidemiol. 2011;21(3):191-6.

33. Dong B, Wang Z, Arnold LW, Song Y, Wang HJ, Ma J. Simplifying the screening of abdominal adiposity in Chinese children with waist-to-height ratio. Am J Hum Biol. 2016;28(6):945-9.

34. Ejtahed HS, Kelishadi R, Qorbani M, Motlagh ME, Hasani-Ranjbar S, Angoorani P, et al. Utility of waist circumference-to-height ratio as a screening tool for generalized and central obesity among Iranian children and adolescents: The CASPIAN-V study. Pediatr Diabetes. 2019;20(5):530-7.

35. Chen G, Yan H, Hao Y, Shrestha S, Wang J, Li Y, et al. Comparison of various anthropometric indices in predicting abdominal obesity in Chinese children: a cross-sectional study. BMC Pediatr. 2019;19(1):127.
