# Supplementary material for: Performance of waist-to-height ratio as a screening tool for identifying cardiometabolic risk in children: a meta-analysis
Source: Diabetol Metab Syndr. 2021 Jun 14;13:66. doi: 10.1186/s13098-021-00688-7 (PMC8201900; doi:10.1186/s13098-021-00688-7)
Supplement: Supplementary file 7 — Additional file 7: Table S5. Pooled results of WHtR screening for CMRs in East-Asian children. [file 13098_2021_688_MOESM7_ESM.docx]

| Outcome | Number of studies | Number of Units | Prevalence | *Q* | *P*-value | *I^2^* | Threshold effect (correlation coefficient) | *P*-value | The proportion of heterogeneity likely due to a threshold effect |
| --- | --- | --- | --- | --- | --- | --- | --- | --- | --- |
| CMR_3_ (1, 2) (3) (4-9) | 9 | 62595 | 0.050 | 698.53 | ＜0.001 | 100 (100-100) | 0.39 | 0.305 | 0.21 |
| CMR_2_ (3) (10-12) (9, 13) | 6 | 38766 | 0.100 | 966.11 | ＜0.001 | 100 (100-100) | -0.01 | 0.985 | 0.05 |
| CMR_1_ (3) (9, 11) | 3 | 14239 | 0.540 | 361.97 | ＜0.001 | 99 (99-100) | -0.21 | 0.867 | 0.00 |
| Elevated FBG (3, 8, 11, 14) | 4 | 11071 | 0.240 | 811.50 | ＜0.001 | 100 (100-100) | 0.97 | 0.031 | 0.82 |
| Elevated BP (1, 3, 8, 15, 16) (9, 11, 17) | 8 | 50191 | 0.140 | 1106.86 | ＜0.001 | 100 (100-100) | 0.95 | <0.001 | 0.91 |
| Dyslipidemia (1, 3, 8, 11, 18) (9) | 12 | 66659 | 0.140 | 1104.40 | ＜0.001 | 100 (100-100) | 0.87 | <0.001 | 0.94 |
| Central obesity (3, 5, 19-21) | 5 | 131164 | 0.190 | 460.60 | ＜0.001 | 100 (99-100) | -0.68 | 0.207 | 0.53 |

**Table S5. Pooled results of WHtR screening for CMRs in East-Asian children**

WHtR: waist-to-height ratio; CMR: cardiometabolic risk factor; CMR_3_: presenting with at least three of CMRs; CMR_2_: presenting with at least two of CMRs; CMR_1_: presenting with at least one of CMRs; FBG: fasting blood glucose; BP, blood pressure.
AUSROC, area under the summary receiver operating characteristic; PLR, positive likelihood ratio; NLR, negative likelihood ratio; DOR, diagnostic odds ratio; CI: confidence interval.
The results of pooled elevated total cholesterol/ triglyceride /low high-density leptin cholesterol/elevated low-density leptin cholesterol and elevated SBP/DBP were not shown.

(Continued)

| Outcomes | AUSROC (95% *CI*) | Sensitivity (95% *CI*) | Specificity (95% *CI*) | PLR (95% *CI*) |
| --- | --- | --- | --- | --- |
| CMR_3_ | 0.92 (0.89, 0.94) | 0.86 (0.71, 0.93) | 0.85 (0.75, 0.91) | 5.60 (3.40, 9.20) |
| CMR_2_ | 0.88 (0.85, 0.90) | 0.85 (0.63, 0.95) | 0.82 (0.76, 0.87) | 4.80 (3.20, 7.10) |
| CMR_1_ | 0.79 (0.75, 0.82) | 0.55 (0.40, 0.70) | 0.87 (0.75, 0.93) | 4.10 (2.00, 8.50) |
| Elevated FBG | 0.62 (0.57, 0.66) | 0.48 (0.24, 0.73) | 0.74 (0.35, 0.93) | 1.80 (0.90, 3.80) |
| evated BP | 0.70 (0.65, 0.74) | 0.49 (0.35, 0.62) | 0.78 (0.68, 0.86) | 2.20 (1.90, 2.60) |
| Dyslipidemia | 0.64 (0.60, 0.68) | 0.44 (0.31, 0.58) | 0.75 (0.63, 0.84) | 1.80 (1.50, 2.00) |
| Central obesity | 0.95 (0.93, 0.97) | 0.91 (0.80, 0.96) | 0.92 (0.89, 0.94) | 10.70 (7.70, 15.00) |

(Continued)

| Outcomes | NLR (95% *CI*) | DOR (95% *CI*) | Correlation Coefficient (95% *CI*) of Deek’s Funnel Plot Asymmetry Test | *t* | *P*-value |
| --- | --- | --- | --- | --- | --- |
| CMR_3_ | 0.17 (0.08, 0.35) | 33.00 (14.00, 75.00) | 58.04 (-41.36, 157.43) | 1.38 | 0.210 |
| CMR_2_ | 0.18 (0.06, 0.52) | 27.00 (7.00, 105.00) | 37.59 (-71.61, 146.79) | 0.96 | 0.393 |
| CMR_1_ | 0.52 (0.36, 0.75) | 8.00 (3.00, 22.00) | -86.16 (-898.11, 725.79) | -1.35 | 0.406 |
| Elevated FBG | 0.71 (0.55, 0.90) | 3.00 (1.00, 6.00) | 25.63 (-45.11, 96.38) | 1.56 | 0.259 |
| Elevated BP | 0.66 (0.56, 0.77) | 3.00 (3.00, 4.00) | 4.36 (-33.52, 42.24) | 0.28 | 0.788 |
| Dyslipidemia | 0.75 (0.65, 0.85) | 2.00 (2.00, 3.00) | 1.85 (-10.83, 14.52) | 0.32 | 0.752 |
| Central obesity | 0.10 (0.04, 0.23) | 110.00 (35.00, 346.00) | 67.14 (-98.68, 232.95) | 1.29 | 0.288 |

**References**

1. Li Y, Zou Z, Luo J, Ma J, Ma Y, Jing J, et al. The predictive value of anthropometric indices for cardiometabolic risk factors in Chinese children and adolescents: A national multicenter school-based study. PLoS One. 2020;15(1):e0227954.

2. Zhang Y, Hu J, Li Z, Li T, Chen M, Wu L, et al. A Novel Indicator Of Lipid Accumulation Product Associated With Metabolic Syndrome In Chinese Children And Adolescents. Diabetes Metab Syndr Obes. 2019;12:2075-83.

3. Dou Y, Jiang Y, Yan Y, Chen H, Zhang Y, Chen X, et al. Waist-to-height ratio as a screening tool for cardiometabolic risk in children and adolescents: a nationwide cross-sectional study in China. BMJ Open. 2020;10(6):e037040.

4. Liu BY, Jiang Rh, Li P, Liu C, Li L. Cutoff Waist-to-height and Waist-to-hip Ratios for Metabolic Syndrome in Chinese Children and Adolescents. Journal of China Medical University. 2017;46(5):434-8,43.

5. Zhou D, Yang M, Yuan ZP, Zhang DD, Liang L, Wang CL, et al. Waist-to-Height Ratio: a simple, effective and practical screening tool for childhood obesity and metabolic syndrome. Prev Med. 2014;67:35-40.

6. Xu T, Liu J, Liu J, Zhu G, Han S. Relation between metabolic syndrome and body compositions among Chinese adolescents and adults from a large-scale population survey. BMC Public Health. 2017;17(1):337.

7. Ma CM, Yin FZ, Liu XL, Wang R, Lou DH, Lu Q. How to Simplify the Diagnostic Criteria of Metabolic Syndrome in Adolescents. Pediatr Neonatol. 2017;58(2):178-84.

8. Nan Zh, Cui L, Cui MH, Xu MH, Jin YH, Fang JN. Relationships of different types of obesity with metabolic syndrome and its components among Han-Chinese adolescents in Yanbian area. Chinese Journal of School Health. 2013;34(4):457-9.

9. Meng Lh, Mi J. The validation of the classification criterion of waist and waist-to-height ratio for cardiometabolic risk factors in Chinese school-age children. Chinese Journal of Evidence Based Pediatrics. 2008;3(5):324-32.

10. Seo JY, Kim JH. Validation of surrogate markers for metabolic syndrome and cardiometabolic risk factor clustering in children and adolescents: A nationwide population-based study. PLoS One. 2017;12(10):e0186050.

11. HOU YP, YANG L, XI B. Comparison of the performance of waist circumference， waist-height ratio， and body mass index in predicting metabolic disorders among children and adolescents. Chinese Journal of Child Health Care. 2018;26(3):239-42,57.

12. Liu XL, Yin FZ, Ma CP, Gao GQ, Ma CM, Wang R, et al. Waist-to-height ratio as a screening measure for identifying adolescents with hypertriglyceridemic waist phenotype. J Pediatr Endocrinol Metab. 2015;28(9-10):1079-83.

13. Dai Y, Fu J, Liang L, Gong C, Xiong F, Liu G, et al. [A proposal for the cutoff point of waist-to-height for the diagnosis of metabolic syndrome in children and adolescents in six areas of China]. Zhonghua Liu Xing Bing Xue Za Zhi. 2014;35(8):882-5.

14. Yuan Y, Xie H, Sun L, Wang B, Zhang L, Han H, et al. A Novel Indicator of Children's Lipid Accumulation Product Associated with Impaired Fasting Glucose in Chinese Children and Adolescents. Diabetes Metab Syndr Obes. 2020;13:1653-60.

15. Wang Y, Liu W, Sun L, Zhang Y, Wang B, Yuan Y, et al. A novel indicator, childhood lipid accumulation product, is associated with hypertension in Chinese children and adolescents. Hypertens Res. 2020;43(4):305-12.

16. Xue J. The predictive effect of obesity-related indicators and blood pressure to height ratio on hypertension among urban school-age children: Shandong University; 2014.

17. Ma CW, Liang YJ, Xi B. Comparison of the performance of waist circumference and waist-height ratio in predicting elevated blood pressure among children and adolescents. Chinese Journal of School Health. 2016;37(10):1445-8.

18. Liu Y, Mi J, Han W, Jin Hf, Du Jb. Analyze the indices of the screening test of hyperlipidemia by Logistic regression analysis and ROC study in children. BASIC & CLINICAL MEDICINE. 2007;27(2):152-6.

19. Fujita Y, Kouda K, Nakamura H, Iki M. Cut-off values of body mass index, waist circumference, and waist-to-height ratio to identify excess abdominal fat: population-based screening of Japanese school children. J Epidemiol. 2011;21(3):191-6.

20. Dong B, Wang Z, Arnold LW, Song Y, Wang HJ, Ma J. Simplifying the screening of abdominal adiposity in Chinese children with waist-to-height ratio. Am J Hum Biol. 2016;28(6):945-9.

21. Chen G, Yan H, Hao Y, Shrestha S, Wang J, Li Y, et al. Comparison of various anthropometric indices in predicting abdominal obesity in Chinese children: a cross-sectional study. BMC Pediatr. 2019;19(1):127.
