# Supplementary material for: Performance of waist-to-height ratio as a screening tool for identifying cardiometabolic risk in children: a meta-analysis
Source: Diabetol Metab Syndr. 2021 Jun 14;13:66. doi: 10.1186/s13098-021-00688-7 (PMC8201900; doi:10.1186/s13098-021-00688-7)
Supplement: Supplementary file 8 — Additional file 8: Table S6. Sensitivity analyses: pooled results of WHtR screening for clusters of CMR with identical components [file 13098_2021_688_MOESM8_ESM.docx]

| Outcome | Number of studies | Number of Units | Prevalence | *Q* | *P*-value | *I^2^* | Threshold effect (correlation coefficient) | *P*-value | The proportion of heterogeneity likely due to a threshold effect |
| --- | --- | --- | --- | --- | --- | --- | --- | --- | --- |
| CMR_3 (1-13)_ | 13 | 52961 | 0.070 | 415.31 | ＜0.001 | 100 (99-100) | 0.04 | 0.905 | 0.01 |

**Table S6. Sensitivity analyses: pooled results of WHtR screening for clusters of CMR with identical components.**

WHtR: waist-to-height ratio; CMR: cardiometabolic risk factor; CMR_3_: presenting with at least three of CMRs (components: elevated fasting blood glucose, low high-density leptin cholesterol, elevated triglyceride, elevated blood pressure, central obesity); AUSROC, area under the summary receiver operating characteristic; PLR, positive likelihood ratio; NLR, negative likelihood ratio; DOR, diagnostic odds ratio; CI: confidence interval.
The result of pooled CMR_1_ was not pooled due to limited original studies.

(Continued)

| Outcomes | AUSROC (95% *CI*) | Sensitivity (95% *CI*) | Specificity (95% *CI*) | PLR (95% *CI*) |
| --- | --- | --- | --- | --- |
| CMR_3_ | 0.90 (0.80, 0.95) | 0.89 (0.82, 0.93) | 0.89 (0.82, 0.93) | 8.00 (4.90, 13.00) |

(Continued)

| Outcomes | NLR (95% *CI*) | DOR (95% *CI*) | Correlation Coefficient (95% *CI*) of Deek’s Funnel Plot Asymmetry Test | *t* | *P*-value |
| --- | --- | --- | --- | --- | --- |
| CMR_3_ | 0.12 (0.06, 0.23) | 69.00 (28.00, 169.00) | -9.25 (-47.94, 29.43) | -0.53 | 0.609 |

**References**

1. Cristine Silva K, Santana Paiva N, Rocha de Faria F, Franceschini S, Eloiza Piore S. Predictive Ability of Seven Anthropometric Indices for Cardiovascular Risk Markers and Metabolic Syndrome in Adolescents. J Adolesc Health. 2020;66(4):491-8.

2. Nan Zh, Cui L, Cui MH, Xu MH, Jin YH, Fang JN. Relationships of different types of obesity with metabolic syndrome and its components among Han-Chinese adolescents in Yanbian area. Chinese Journal of School Health. 2013;34(4):457-9.

3. Perona JS, Schmidt-RioValle J, Rueda-Medina B, Correa-Rodriguez M, Gonzalez-Jimenez E. Waist circumference shows the highest predictive value for metabolic syndrome, and waist-to-hip ratio for its components, in Spanish adolescents. Nutr Res. 2017;45:38-45.

4. Zhou D, Yang M, Yuan ZP, Zhang DD, Liang L, Wang CL, et al. Waist-to-Height Ratio: a simple, effective and practical screening tool for childhood obesity and metabolic syndrome. Prev Med. 2014;67:35-40.

5. Aguirre PF, Coca A, Aguirre MF, Celis G. Waist-to-height ratio and sedentary lifestyle as predictors of metabolic syndrome in children in Ecuador. Hipertens Riesgo Vasc. 2017.

6. Ma CM, Yin FZ, Liu XL, Wang R, Lou DH, Lu Q. How to Simplify the Diagnostic Criteria of Metabolic Syndrome in Adolescents. Pediatr Neonatol. 2017;58(2):178-84.

7. Xu T, Liu J, Liu J, Zhu G, Han S. Relation between metabolic syndrome and body compositions among Chinese adolescents and adults from a large-scale population survey. BMC Public Health. 2017;17(1):337.

8. Oliveira RG, Guedes DP. Performance of anthropometric indicators as predictors of metabolic syndrome in Brazilian adolescents. BMC Pediatr. 2018;18(1):33.

9. Liu BY, Jiang Rh, Li P, Liu C, Li L. Cutoff Waist-to-height and Waist-to-hip Ratios for Metabolic Syndrome in Chinese Children and Adolescents. Journal of China Medical University. 2017;46(5):434-8,43.

10. Arsang-Jang S, Kelishadi R, Esmail Motlagh M, Heshmat R, Mansourian M. Temporal Trend of Non-Invasive Method Capacity for Early Detection of Metabolic Syndrome in Children and Adolescents: A Bayesian Multilevel Analysis of Pseudo-Panel Data. Ann Nutr Metab. 2019;75(1):55-65.

11. Vasquez F, Correa-Burrows P, Blanco E, Gahagan S, Burrows R. A waist-to-height ratio of 0.54 is a good predictor of metabolic syndrome in 16-year-old male and female adolescents. Pediatr Res. 2019;85(3):269-74.

12. Benmohammed K, Valensi P, Benlatreche M, Nguyen MT, Benmohammed F, Paries J, et al. Anthropometric markers for detection of the metabolic syndrome in adolescents. Diabetes Metab. 2015;41(2):138-44.

13. Zhang Y, Hu J, Li Z, Li T, Chen M, Wu L, et al. A Novel Indicator Of Lipid Accumulation Product Associated With Metabolic Syndrome In Chinese Children And Adolescents. Diabetes Metab Syndr Obes. 2019;12:2075-83.
